# Supplementary material for: Living Neurons with Tau Filaments Aberrantly Expose Phosphatidylserine and Are Phagocytosed by Microglia
Source: Cell Rep. Author manuscript; Available in PMC 2018 Sep 28. (PMC6161320; doi:10.1016/j.celrep.2018.07.072)
Supplement: 1 [file NIHMS1505383-supplement-1.pdf]

Cell Reports, Volume *24*

Supplemental Information

Living Neurons with Tau Filaments Aberrantly Expose Phosphatidylserine and Are Phagocytosed by Microglia

Jack Brelstaff, Aviva M. Tolkovsky, Bernardino Ghetti, Michel Goedert, and Maria Grazia Spillantini

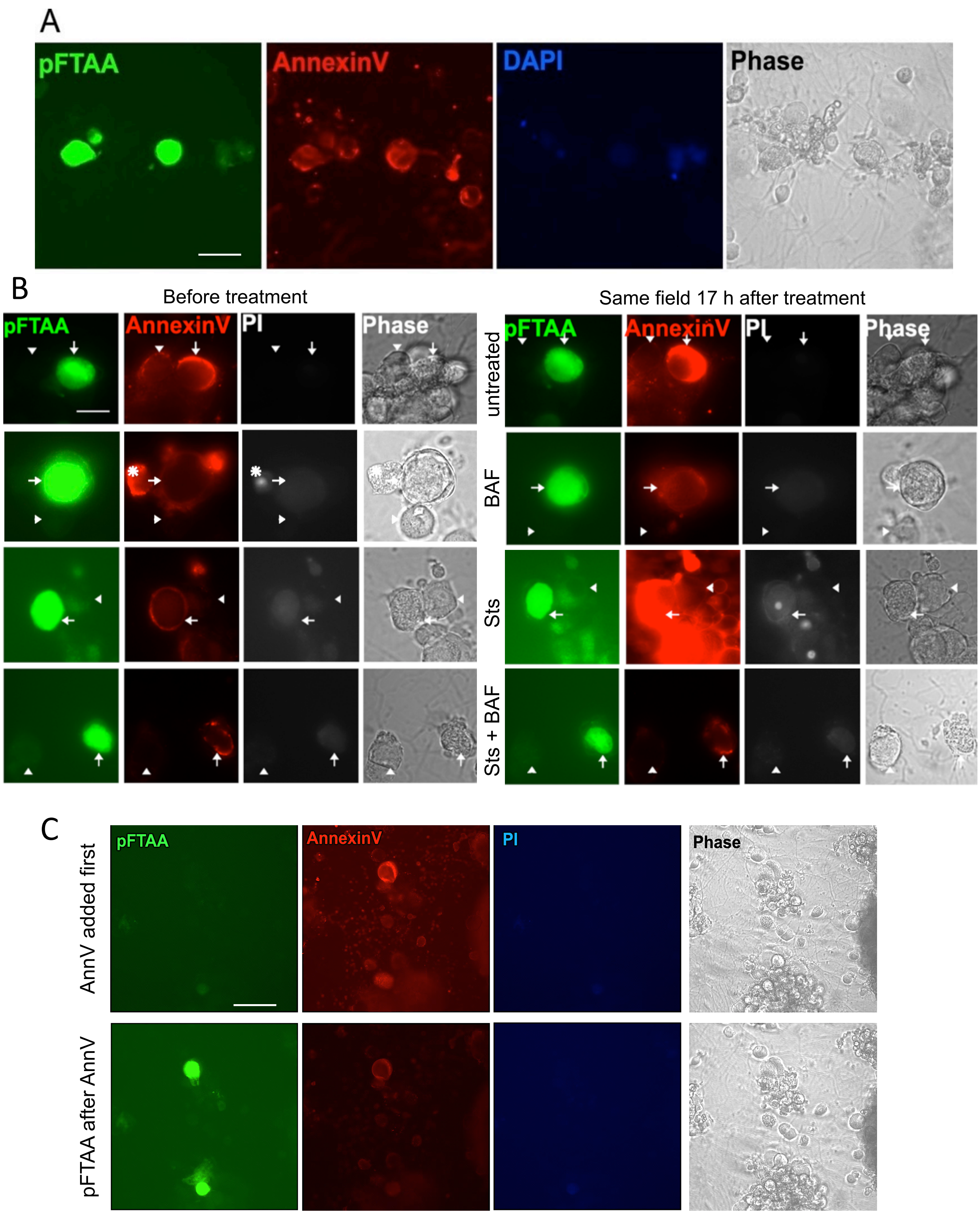

Figure S1. Neurons with tau aggregates are living neurons. Related to Figure 1.
(A) Living pFTAA+ve neurons displaying PtdSer are not stained with the live-cell-impermeable nuclear stain DAPI.
(B) The pan-caspase inhibitor Boc-Asp-FMK (BAF) prevents neurons treated with 250 nM Staurosporine (Sts) for 17 hours from displaying PtdSer during apoptosis and becoming PI+ve due to secondary necrosis, but it has no effect on PtdSer exposure in pFTAA+ve neurons.
(C) AnnV staining of living neurons with tau aggregates is enhanced independently of pFTAA binding. Scale bars: A = 50 µm, B = 25 µm, C=50 µm.

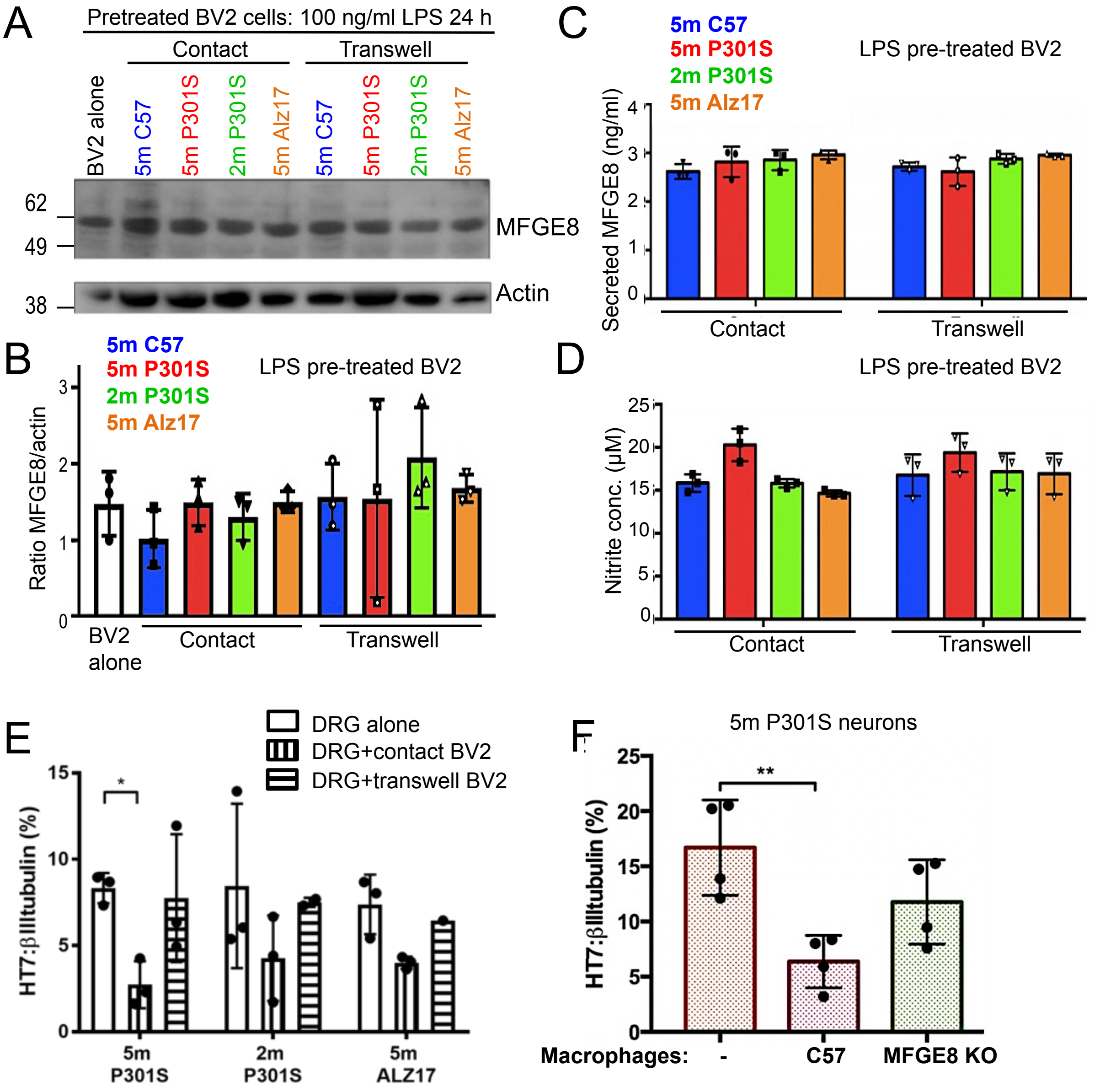

Figure S2. LPS pretreated BV2 cells produce the same amounts of MFGE8 and NO irrespective of neuron source or culture conditions but still favour 5m P301S-tau+ve neurons for phagocytosis. Related to Figure 3.

(A) Representative immunoblot showing that BV2 cells treated with 100 ng/ml LPS for 24 h and washed before culturing alone or in co-culture with neurons for 4 days express similar elevated amounts of MFGE8 irrespective of neuronal source and regardless of whether the BV2 cells were in contact with the neurons or in transwells.

(B) Quantification of MFGE8 expression from 3 independent experiments.

(C) The amount of MFGE8 secreted from BV2 cells pretreated with 100 ng/ml LPS for 24 h and co-cultured with neurons results in no differences, as described in A.

(D) Treatment of BV2 microglia with LPS as described in (A) induced similar amounts of NO (around 15 μM nitrite equivalents) to those induced by neurons from 5m P301S-tau mice.

(E) The percentage of HT7+ve neurons vs. total βIIItubulin+ve neurons remaining after neurons were cultured with LPS-pre-treated BV2 microglia in contact or via transwells for 4 days. DRG neurons from 5m P301S-tau mice are lost from cultures in contact with LPS pre-treated BV2 microglia (*=$p<0.05$) but not when BV2 microglia are in transwells. No HT7+ve neurons are lost when BV2 microglia are cultured with neurons from 2m P301S-tau mice or 5m Alz17 mice. Mean ± SD, n=3 independent experiments. 2-way ANOVA, Bonferroni corrected.

(F) Bone marrow-derived macrophages (BMDM) cultured from C57 mice in contact with DRG neurons from 5m P301S-tau mice induced significant loss of htau+ve neurons ($p<0.01$), but there was no significant loss of neurons when BMDMs were prepared from MFGE8 knockout mice, 1-way ANOVA, Bonferroni corrected.

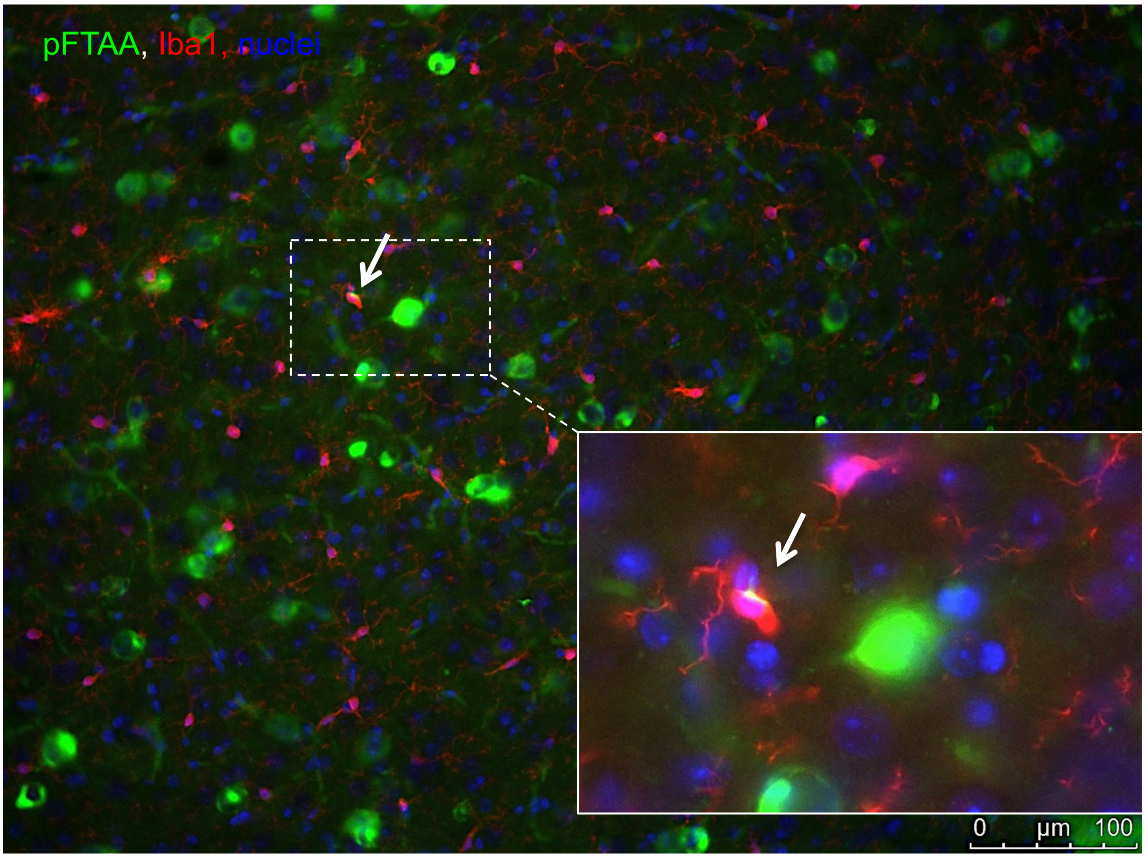

Figure S3. pFTAA+ve tau inside a microglial cell in the motor cortex. Related to Figure 4.
Section (25 µm thick) through the level of the primary motor cortex M1 (interaural 5.12 mm, Bregma 1.32 mm) stained with pFTAA (green), anti-Iba1 (red), and Dapi (blue).

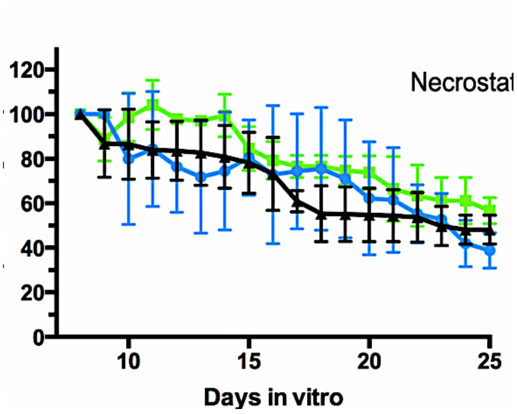

Figure S4. Loss of P301S-tau neurons is not due to necroptosis. Related to Figure 4.

The rate of pFTAA+ve neuron loss is not altered when DRG cultures from 5m P301S-tau are maintained in the presence of 10 µM necrostatin-1 (Nec1) or 10 µM of the inactive analogue (Nec1i). DRG neurons from 5m P301S-tau mice were maintained in culture for 8 days before addition of the inhibitors. Each day, the number of pFTAA +ve neurons remaining in the same fields was counted. Each point shows mean ± SD, n=3 independent experiments.

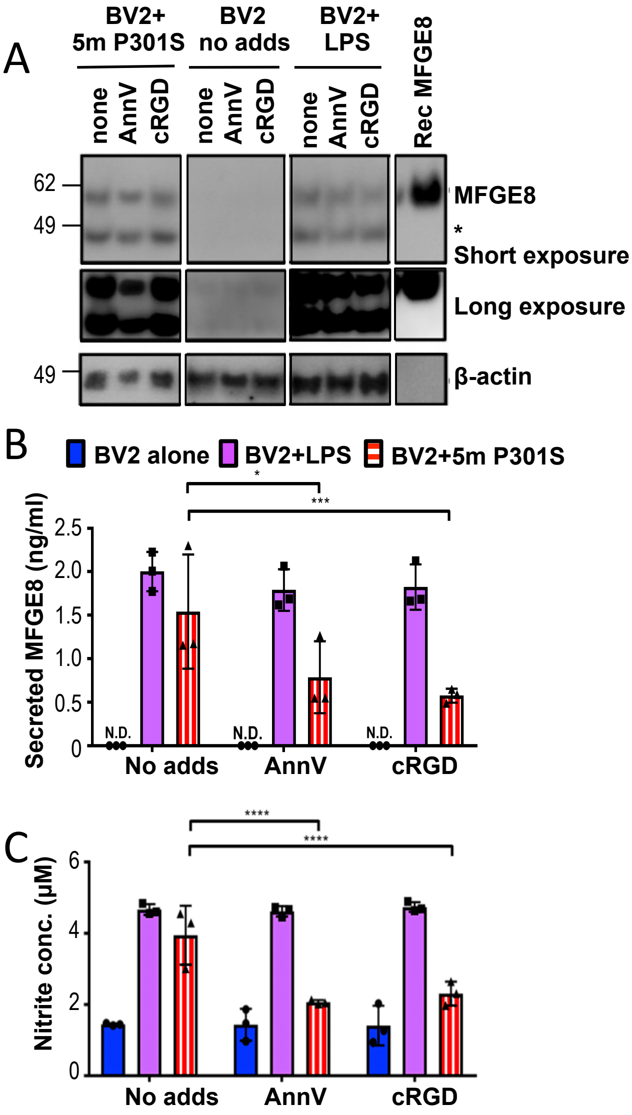

Figure S5. AnnV or RGD do not inhibit MFGE8 or NO release from LPS-treated BV2 cells. Related to Figure 5.

(A-C) AnnV or cRGD do not inhibit MFGE8 expression (D), MFGE8 secretion (E) or NO production (F) by BV2 cells stimulated with LPS, nor inhibit the intracellular amounts of MFGE8 expressed when BV2 cells are cultured with neurons from 5m P301S-tau mice. Both drugs do inhibit MFGE8 and NO secretion in the co-cultures. * indicates either another isoform of MFGE8 or a breakdown product. Mean ± SD, n=3 independent experiments analysed by 1-way ANOVA, Bonferroni corrected.
