# Supplementary material for: Living Neurons with Tau Filaments Aberrantly Expose Phosphatidylserine and Are Phagocytosed by Microglia
Source: Cell Rep. Author manuscript; Available in PMC 2018 Sep 28. (PMC6161320; doi:10.1016/j.celrep.2018.07.072)
Supplement: 2 [file NIHMS1505383-supplement-2.pdf]

Cell Reports

Report

Living Neurons with Tau Filaments Aberrantly Expose Phosphatidylserine and Are Phagocytosed by Microglia

Graphical Abstract

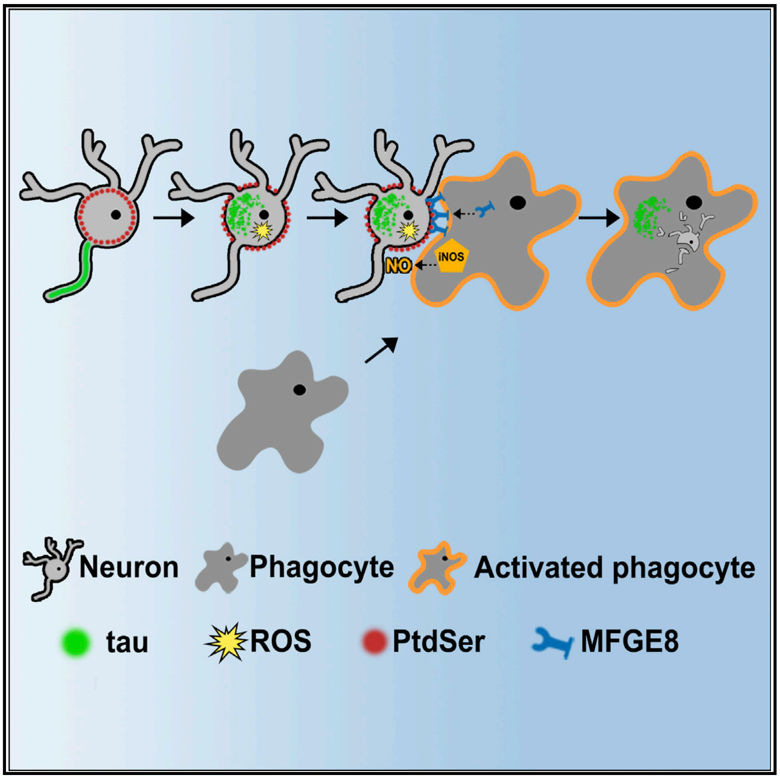

Highlights

- Live neurons with P301S-tau filaments expose phosphatidylserine due to elevated ROS
- Co-cultured microglia phagocytose these neurons by releasing MFGE8 and NO
- Inhibiting MFGE8 and NO production blocks phagocytosis and prevents neuron death
- Elevated MFGE8 correlates with tau inclusion load in frontotemporal dementias

Authors

Jack Brelstaff, Aviva M. Tolkovsky, Bernardino Ghetti, Michel Goedert, Maria Grazia Spillantini

Correspondence

amt1004@cam.ac.uk (A.M.T.), mgs11@cam.ac.uk (M.G.S.)

In Brief

Brelstaff et al. report that live neurons containing aggregated tau externalize phosphatidylserine, activate microglia, and are phagocytosed. Preventing key steps in this pathway rescues living neurons. A similar phagocytic signal is found in human tauopathies. The authors propose that inhibiting phagocytosis may spare neurons with tau aggregates.

Brelstaff et al., 2018, Cell Reports *24*, 1939–1948
August 21, 2018 © 2018 The Authors.
https://doi.org/10.1016/j.celrep.2018.07.072

OPEN ACCESS
CellPress

Living Neurons with Tau Filaments Aberrantly Expose Phosphatidylserine and Are Phagocytosed by Microglia

Jack Brelstaff,[1] Aviva M. Tolkovsky,[1,*] Bernardino Ghetti,[2] Michel Goedert,[3] and Maria Grazia Spillantini[1,4,*]
[1]Department of Clinical Neurosciences, Clifford Allbutt Building, University of Cambridge, Cambridge, CB2 0AH, UK
[2]Department of Pathology and Laboratory Medicine, Indiana University, Indianapolis, IN, USA
[3]Medical Research Council Laboratory of Molecular Biology, Cambridge CB2 0QH, UK
[4]Lead Contact
*Correspondence: amt1004@cam.ac.uk (A.M.T.), mgs11@cam.ac.uk (M.G.S.)
https://doi.org/10.1016/j.celrep.2018.07.072

SUMMARY

Tau protein forms insoluble filamentous inclusions that are closely associated with nerve cell death in many neurodegenerative diseases. How neurons die in these tauopathies is unclear. We report that living neurons with tau inclusions from P301S-tau mice expose abnormally high amounts of phosphatidylserine because of the production of reactive oxygen species (ROS). Consequently, co-cultured phagocytes (BV2 cells or primary microglia) identify and phagocytose the living neurons, thereby engulfing insoluble tau inclusions. To facilitate engulfment, neurons induce contacting microglia to secrete the opsonin milk-fat-globule EGF-factor-8 (MFGE8) and nitric oxide (NO), whereas neurons with tau inclusions are rescued when MFGE8 or NO production is prevented. MFGE8 expression is elevated in transgenic P301S-tau mouse brains with tau inclusions and in tau inclusion-rich brain regions of several human tauopathies, indicating shared mechanisms of disease. Preventing phagocytosis of living neurons will preserve them for treatments that inhibit tau aggregation and toxicity.

INTRODUCTION

The assembly of tau protein into abnormal inclusions underlies many human neurodegenerative diseases (Spillantini and Goedert, 2013), but how neurons die in tauopathies is still unknown. Transgenic mice that express neuron-specific human mutant 0N4R P301S-tau reproduce much of the tau pathology observed in a family with frontotemporal dementia due to a P301S-tau mutation (Bugiani et al., 1999), with neurons in the central and peripheral nervous systems developing filamentous tau inclusions and progressive neurodegeneration between 3 and 5 months of age (Allen et al., 2002; Mellone et al., 2013). Peripheral neurons are also affected in human tauopathies (Kawasaki et al., 1987; Nishimura et al., 1993), making them a relevant model of disease. We reported that the pentameric oligothiophene dye pFTAA specifically detects filamentous tau aggregates in dorsal root ganglion (DRG) neurons from P301S-tau mice (Brelstaff et al., 2015a, 2015b), enabling investigation of how tau aggregates may lead to cell death. Observing pFTAA$^+$ cultured DRG neurons showed that they are slowly removed, without showing signs of apoptosis or necroptosis (Brelstaff et al., 2015a). Such slow kinetics accord with phagocytic cell death of live neurons by microglia (Neher et al., 2011). Glial cells, particularly microglia, are thought to be crucial in neurodegeneration (Salter and Stevens, 2017; Spangenberg and Green, 2017). Microglial activation has been associated with tau aggregation in frontotemporal dementia (FTDP-17T) (Bellucci et al., 2011) and P301S-tau mouse brains (Bellucci et al., 2004) and has also been implicated in tau spreading through phagocytosis (Bolós et al., 2016; Maphis et al., 2015).

Death of cells through phagocytosis occurs extensively (Brown et al., 2015) and is required for programmed cell death in *C. elegans* (Johnsen and Horvitz, 2016). Most studies of neuronal cell death through microglial phagocytosis have relied on the induction of phagocytic activity by inflammatory signals (Brown and Neher, 2014). Inflammatory microglia instigate living neurons to expose the "eat me" signal phosphatidylserine (PS) and perform phagocytosis through release of opsonins (e.g., MFGE8). MFGE8 simultaneously binds target-exposed PS (Hanayama et al., 2002) and phagocytic $\alpha v\beta 3$ vitronectin receptors, causing cytoskeletal rearrangements that facilitate target engulfment. Whether non-apoptotic exposure of PS occurs on diseased neurons and whether it activates microglial inflammation and phagocytosis are unknown.

We have investigated how tau inclusion-bearing neurons die, showing that live neurons with aggregated tau produce sufficient reactive oxygen species (ROS) to externalize PS and activate microglial phagocytosis. Preventing key steps in this pathway leads to the rescue of living neurons.

RESULTS

Living Neurons with Tau Inclusions Display PS through a ROS-Dependent Mechanism

Neurons cultured from 5-month-old P301S-tau mice (P301S mice) were probed with the PS-binding protein annexin V (AnnV-Alexa Fluor 647) and cell-impermeable nuclear dyes. Living DRG neurons with pFTAA$^+$ tau inclusions displayed significantly more externalized PS compared with pFTAA$^-$ neurons

This is an open access article under the CC BY license (http://creativecommons.org/licenses/by/4.0/).

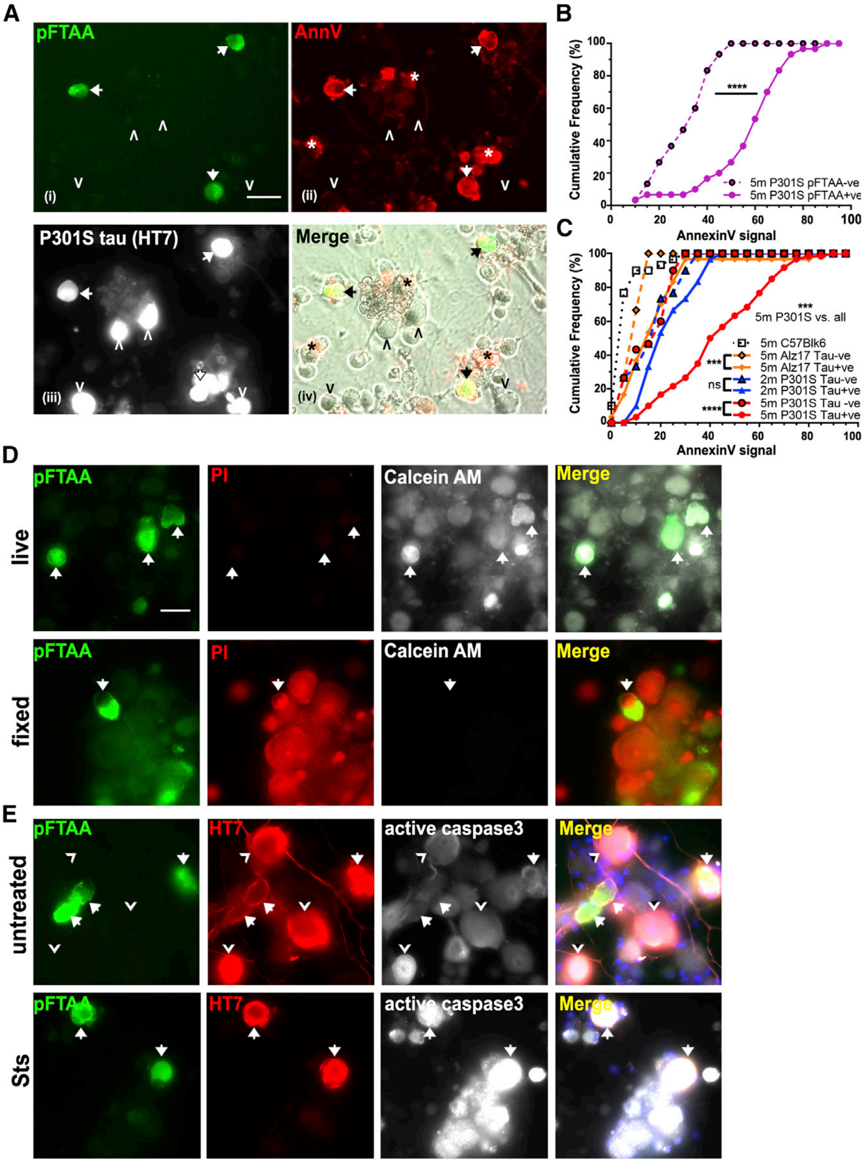

Figure 1. Living Neurons with pFTAA$^+$ Tau Filaments Aberrantly Expose PS by a Reversible ROS-Dependent Mechanism

(A) Living DRG neurons from 5-month-old P301S mice with filamentous tau aggregates stained with (i) pFTAA (green) and (ii) AnnV-647 (red) (arrows) (asterisk denotes dead cell debris); (iii) same neurons fixed and stained for human tau (HT7 antibody). pFTAA$^-$/HT7$^+$ neurons do not stain with AnnV-647 (arrowheads). (iv) Nontransgenic (HT7$^-$) neurons are AnnV$^-$; images (i) and (ii) merged with phase contrast. Scale bar, 25 μm.

(B) Higher signal intensities of AnnV-647 binding to pFTAA$^+$ versus pFTAA$^-$ neurons in live cultures from 5-month-old P301S mice (****$p < 0.0001$). Cumulative frequency plot, 30 neurons per culture, n = 3 independent experiments. Kolmogorov-Smirnov test.

(C) Cumulative frequency plot comparing AnnV-647 binding intensity values of HT7$^+$ and HT7$^-$ neurons in the same cultures from 5-month-old P301S mice (***$p < 0.001$), 2-month-old P301S-mice (not significant [n.s.]), 5-month-old Alz17 mice (***$p < 0.001$), and HT7$^-$ 5-month-old wild-type C57 mice. AnnV staining of HT7$^+$ neurons from 5-month-old P301S mice is significantly more intense than all others (***$p < 0.001$). Thirty neurons, n = 3 independent experiments. Kolmogorov-Smirnov test with Bonferroni correction.

(D) pFTAA$^+$ neurons from 5-month-old P301S mice are living cells. Top row: calcein-AM (white) and PI (red) staining showing exclusion of PI and retention of fluorescent calcein. Bottom row: after fixation, PI stains all cells, and there is no calcein retention. Data representative of n = 3 independent experiments. Scale bar, 30 μm.

(E) pFTAA$^+$ neurons do not display PS, because of activation of caspase-3. Top row: pFTAA$^+$ (green, arrows) and HT7$^+$ (red)/pFTAA$^-$ neurons (arrowheads) showing basal intensities of active caspase-3 staining (white). Bottom row: staurosporine (Sts) induces high levels of active caspase-3 in all the cells.

that also expressed P301S-tau (detected with anti-human tau HT7; Figures 1A and 1B; $p < 0.0001$). PS exposure was highest in cultures containing P301S-tau$^+$ neurons from 5-month-old mice, while significantly lower AnnV labeling was present in DRG neurons cultured from 5-month-old C57BL/6 (C57) control mice, tau$^+$ or tau$^-$ neurons from 2-month-old P301S mice, which express hyperphosphorylated forms of P301S-tau but do not contain filamentous tau aggregates (Delobel et al., 2008; Mellone et al., 2013), and tau$^+$ or tau$^-$ neurons from 5-month-old Alz17 mice that express wild-type 2N4R human tau but do not develop tau aggregates (Brelstaff et al., 2015a; Probst et al., 2000) (Figure 1C; $p < 0.001$ 5-month-old P301S-tau HT7$^+$ versus all others).

pFTAA/AnnV$^+$ neurons were viable, as assessed by these criteria: (1) impermeability to nuclear dyes (PI, Figure 1D; DAPI, Figure S1A), in contrast to strong staining of fixed/permeable cells; (2) retention of calcein-AM, similar to pFTAA$^-$ neurons, in contrast to fixed/permeable cells showing no calcein-AM labeling (Figure 1D); and (3) no active caspase-3, which drives PS exposure on apoptotic cells (Nagata et al., 2016) (Figure 1E), in contrast to caspase-3-dependent exposure of PS on all neurons exposed to pro-apoptotic staurosporine (Sts; 250 nM) (Figure 1E). Also, only Sts-induced PS exposure, but not that induced by P301S-tau, was inhibited by the pan-caspase inhibitor Boc-Asp(*O*-methyl)-FMK (BAF) (Figure S1B). Staining with pFTAA was not the cause of PS exposure, as enhanced AnnV staining intensity was present prior to pFTAA application (Figure S1C). Thus, pFTAA$^+$ neurons with exposed PS are alive.

To investigate what drives PS exposure, we measured ROS production, as ROS can activate PS transporters (Sarkar et al.,

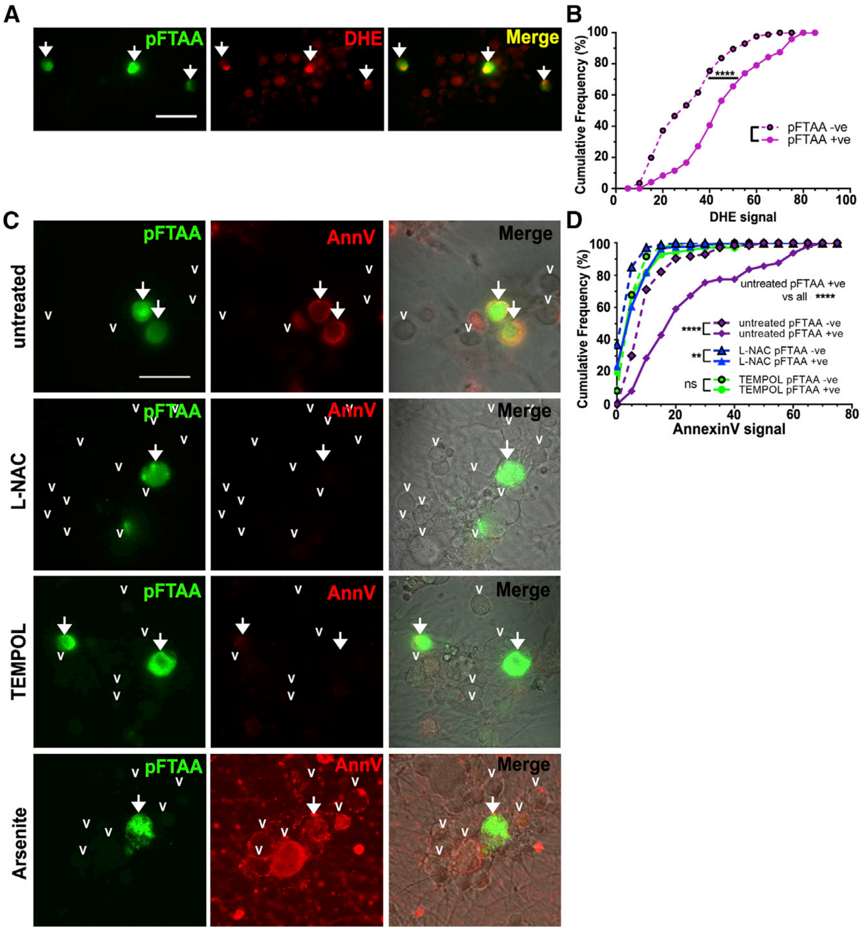

Figure 2. Tau Aggregates Cause PS Externalization through a ROS-Dependent Mechanism

(A and B) Representative image (A) of DRG neurons from 5-month-old P301S mice showing higher intensity of nuclear oxidized DHE staining (red) in pFTAA$^+$ (arrows) versus pFTAA$^-$ neurons. Scale bar, 25 μm. (B) Cumulative frequency plot quantifying DHE fluorescence intensities for pFTAA$^+$ and pFTAA$^-$ DRG neurons; ≥30 neurons per one culture from n = 3 independent experiments. Kolmogorov-Smirnov test. ****$p < 0.0001$.

(C) Loss of AnnV binding to pFTAA$^+$ DRG neurons (arrows) from 5-month-old P301S mice treated with 5 mM NAC or 5 mM TEMPOL for 2 days before washing and staining live with AnnV-647. Arrowheads indicate pFTAA$^-$ neurons. Pro-oxidant arsenite (0.5 mM, 15 min) causes intense AnnV-647 staining in all neurons without any cell death (PI$^-$). Scale bar, 30 μm.

(D) Cumulative frequency plots quantifying AnnV-647 fluorescence intensity values for pFTAA$^+$ and pFTAA$^-$ DRG neurons treated as in (C); ≥30 neurons from n = 3 independent experiments. Kolmogorov-Smirnov test, Bonferroni corrected. Comparison of pFTAA$^+$ values with all others, ****$p < 0.0001$. Values comparing pFTAA$^+$ versus pFTAA$^-$: untreated, ****$p < 0.0001$; NAC, **$p < 0.01$; TEMPOL, n.s.

2015; Schreiber et al., 2018), and oxidized PS is a powerful pro-phagocytic signal (Tyurin et al., 2014). DRG neurons with P301S-tau aggregates displayed significantly higher dihydroethidium (DHE) fluorescence compared with pFTAA$^-$ neurons in the same cultures (Figures 2A and 2B; $p < 0.0001$), indicating higher ROS production. Moreover, PS exposure was ROS dependent, as preincubation with the antioxidants N-acetyl-L-cysteine (NAC) or TEMPOL for 48 hr reduced AnnV binding values compared to untreated pFTAA$^+$ neurons (Figure 2C; $p < 0.0001$, untreated pFTAA$^+$ neurons vs all). In contrast, the pro-oxidant arsenite induced rapid PS exposure in the absence of cell permeabilization or death in all neurons (Figure 2C). Thus, ROS production associated with tau aggregates is a component of the signaling pathway that leads to PS externalization in P301S-tau neurons but is insufficient to cause cell death, unlike ROS-driven ferroptosis/oxytosis (Tan et al., 2001; Yang and Stockwell, 2016).

Neurons with P301S-Tau Inclusions Induce BV2 Cells and Microglia to Secrete MFGE8 and Nitric Oxide

To investigate if cultures containing neurons that display PS would activate phagocytes, microglial-derived BV2 cells were co-cultured in contact with DRG neurons for 4 days before the cells and media were analyzed for nitric oxide (NO) and MFGE8 production, MFGE8 having been shown to be a key opsonin via PS binding in the nervous system (Boddaert et al., 2007; Kranich et al., 2010; Neher et al., 2011). DRG neurons from 5-month-old P301S mice induced a significant increase in the expression and secretion of MFGE8 (Figures 3A–3C) specifically in cultures containing neurons with P301S-tau aggregates, as no increase in its expression was detected when BV2 cells were co-cultured with DRG neurons from 5-month-old Alz17 or C57 mice or 2-month-old P301S mice. Importantly, DRG neurons cultured from 5-month-old P301S mice also induced the release of MFGE8 from co-cultured primary microglia (containing 90% microglia and 10% astrocytes) from C57 mice, MFGE8 concentration being ~6-fold higher than that measured from equivalent numbers of BV2 cells (Figure 3E). No MFGE8 was detected in medium conditioned by microglia alone or microglia from negative-control MFGE8 knockout (KO) mice (Silvestre et al., 2005).

NO was also significantly elevated (~3-fold) in medium conditioned by neurons from 5-month-old P301S mice co-cultured with BV2 cells (Figure 3D), but no increase was detected in co-cultures of BV2 cells with neurons from 5-month-old Alz17 or C57 mice or 2-month-old P301S mice. More NO was also released when neurons from 5-month-old P301S mice were co-cultured with primary microglia from C57 mice, but there was no increase in its release from MFGE8 KO microglia (Figure 3F), suggesting a mutual mechanism of induction between NO- and MFGE8-initiated signaling pathways. Notably, no

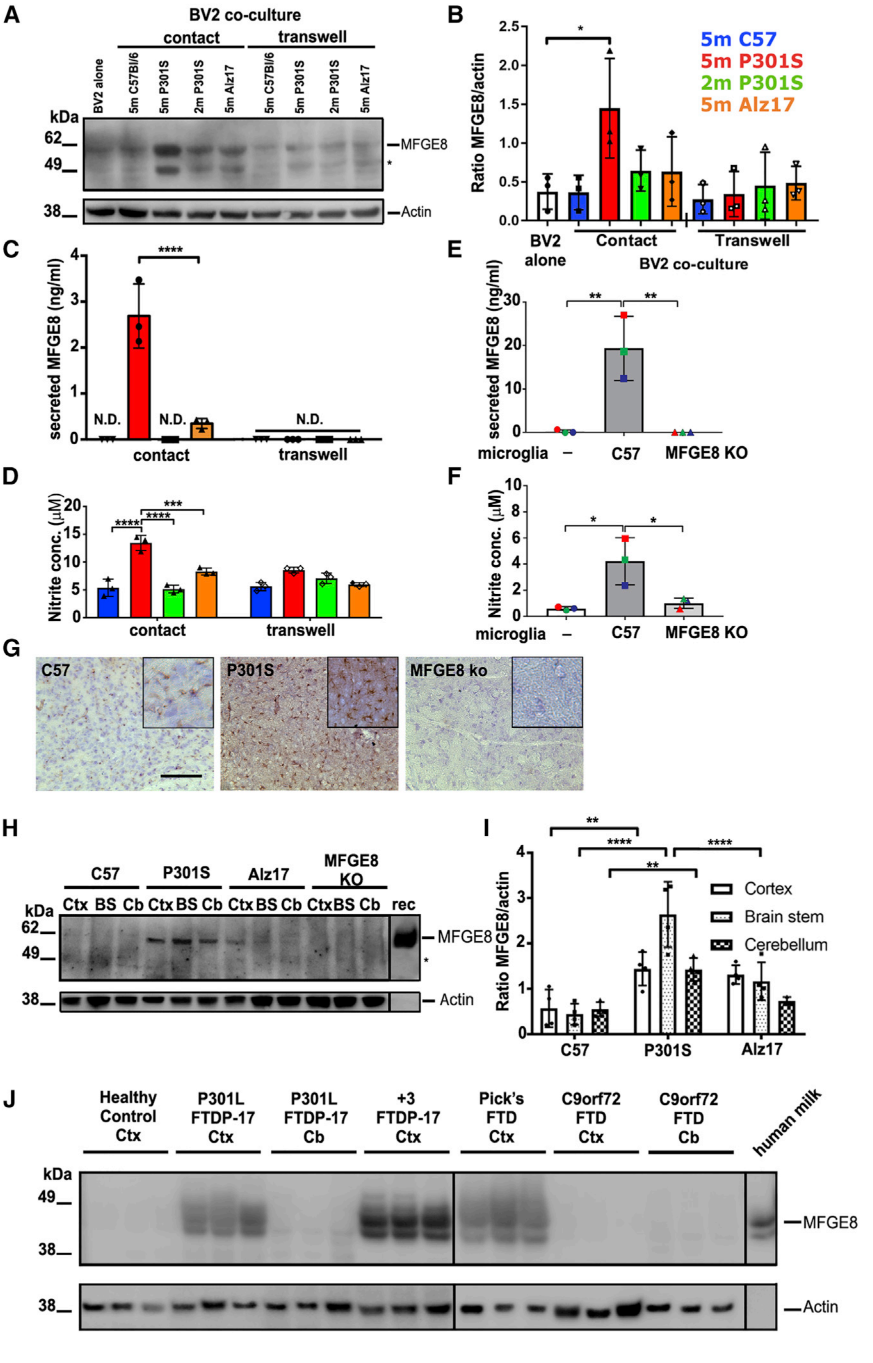

Figure 3. MFGE8 and NO Are Produced and Secreted by Co-cultured BV2 Microglia Only When Co-cultured in Contact with pFTAA$^+$ Neurons

(A and B) Representative blot (A) of MFGE8 in BV2 cells lysates cultured alone or co-cultured in direct contact, or via a transwell, with DRG neurons from 5-month-old P301S-tau, 5-month-old C57, 5-month-old Alz17 mice, or 2-month-old P301S mice. Asterisk: either another isoform of MFGE8 or a breakdown product. (B) Densitometry of MFGE8 expression normalized to β-actin; significantly more MFGE8 is produced only in contact co-cultures containing pFTAA$^+$ neurons. Mean ± SD, n = 3 independent experiments (*$p < 0.05$), two-way ANOVA, Dunnett's correction.

(C and D) Elevated MFGE8 (C) or NO (D) in medium conditioned by BV2 cells co-cultured in contact with DRG neurons from 5-month-old P301S mice compared with 5-month-old Alz17, 5-month-old C57, or 2-month-old P301S mice (MFGE8: ****$p < 0.0001$ versus all; NO: ****$p < 0.0001$ versus 5-month-old C57 or 2-month-old P301S mice; ***$p < 0.001$ versus 5-month-old Alz17 mice). N.D., none detected. Mean ± SD, n = 3 independent experiments, two-way ANOVA, Bonferroni corrected.

(E and F) Elevated MFGE8 (E) or NO (F) in medium conditioned by primary microglia from C57 mice co-cultured in contact with DRG neurons from 5-month-old P301S mice; microglia from MFGE8 KO mice are negative controls. Mean ± SD, n = 3 independent microglial preparations, one-way ANOVA, **$p < 0.01$ (MFGE8), *$p < 0.05$ (NO).

(G) Increased MFGE8 immunostaining intensity in frontal motor cortex of 5-month-old P301S mice compared with 5-month-old C57 mice; MFGE8 KO mouse is negative control; 25 μm section at interaural 5.12 mm, bregma 1.32 mm; brown, DAB; blue, cresyl violet. Scale bar, 130 μm. Inset, 65 μm.

(H and I) Elevated MFGE8 (H) in 5-month-old P301S-tau brains. Lysates from cortex (Ctx), brain stem (BS), and cerebellum (Cb) of 5-month-old C57, 5-month-old P301S-tau, and 5-month-old Alz17 mice probed with anti-MFGE8; MFGE8 KO brain lysate is negative control. rec, recombinant mouse MFGE8. (I) Densitometry of MFGE8 expression normalized to β-actin. Significantly higher MFGE8 expression in P301S versus C57BL/6 Ctx (**$p < 0.01$), BS (****$p < 0.0001$), Cb (**$p < 0.01$), and P301S versus ALz17 BS (****$p < 0.0001$). Mean ± SD, n = 3 independent preparations, two-way ANOVA, Bonferroni corrected.

(J) Elevated MFGE8 expression in brain extracts from cortex (Ctx) of FTDP-17T patients with two different *MAPT* mutations (P301L, +3) or sporadic Pick's disease but not in extracts from patients with C9orf72 hexanucleotide expansions and TDP-43 aggregates. No expression is found in the cerebellum, where tau pathology is absent in all cases. Human milk MFGE8 is a positive control.

increase in MFGE8 in BV2 cell lysates or in released MFGE8 or NO was detected when BV2 cells were co-cultured in transwell inserts regardless of neuronal origins (Figures 3A–3D), indicating a requirement for close contact between neurons and microglia to achieve activation of NO and MFGE8 production.

To assess the intensity and the specificity of stimulation by pFTAA$^+$ neurons, BV2 cells were treated with 100 ng/mL lipopolysaccharide (LPS) for 24 hr and washed prior to their addition to the various neuronal cultures (Figure S2). LPS induced similarly elevated amounts of MFGE8 and NO regardless of neuron source or placement of BV2 cells in contact or transwell inserts. Thus, cultures of neurons with tau inclusions that expose PS provide a microglial stimulus that is as robust as that of LPS, assessed by MFGE8 and NO production.

To demonstrate the relevance of the results from the culture systems to the *in vivo* conditions, brain sections from C57 and P301S mice were stained for the presence of MFGE8 and protein extracts from cortex, brain stem, and cerebellum were analyzed for MFGE8 expression. Less MFGE8 immunoreactivity was evident in the cortex of C57 mice compared with that of P301S mice, though some immunostaining was present in the C57 brains compared with MFGE8 KO controls (Figure 3G). Similarly, extracts from the brains of 5-month-old P301S mice contained significantly higher amounts of MFGE8 compared with lysates from age-matched C57 or Alz17 mice band intensities being slightly higher than background staining in MFGE8 KO lysates (Figures 3H and 3I). Expression of MFGE8 was especially prominent in the brain stem, in which a high number of neurons with tau aggregates is found (Allen et al., 2002).

MFGE8 Expression Is Elevated in the Frontal Cortex of Human Tauopathies

To determine whether a similar pattern of activation occurs in human tauopathies, we compared the expression of MFGE8 in brain extracts from the frontal cortex of healthy human controls and three cases each of inherited tauopathies (P301L and +3 mutations in *MAPT*; Hutton et al., 1998; Mirra et al., 1999; Spillantini et al., 1997) and three cases of sporadic Pick's disease (case details in STAR Methods). Increased MFGE8 expression was found in the cortical extracts of all human tauopathy cases compared with healthy controls (Figure 3J). MFGE8 was barely detected in the cerebellum of P301L-tau cases, an area with little or no tau pathology. We also examined brain extracts from C9orf72 cases containing TDP-43 pathology in the cortex but little tau, as detected by antibody AT8. No increase in MFGE8 was found in the cortex or cerebellum of C9orf72 cases, consistent with the specific expression of MFGE8 in tauopathies and strengthening the relevance of our findings in P301S mice for human disease.

Phagocytes Engulf Neurons with Tau Inclusions that Expose PS

We next investigated if phagocytes specifically engulf P301S-tau^{+} neurons with tau inclusions. Figure 4A shows that when neurons were cultured in contact with BV2 cells, more than 50% of the HT7^{+} neurons were lost after 4 days ($p < 0.001$). There was no loss of HT7^{+} neurons when BV2 cells were co-cultured in a transwell, showing that physical contact was necessary for neuronal loss. In contrast, no HT7^{+} neuron loss was found when BV2 cells were co-cultured with neurons from 5-month-old Alz17 mice or 2-month-old P301S mice. The extent of phagocytosis that followed BV2 activation by neurons was similar to that produced by LPS, in agreement with the similar amounts of MFGE8 and NO produced by both stimuli (Figure S2E; $p < 0.05$). Importantly, microglia from the cortex of neonatal C57 mice caused a similar extent of neuronal loss as BV2 cells (Figure 4B; $p < 0.05$), whereas no neuronal loss occurred with microglia from MFGE8 KO mice (Figure 4B). Macrophages prepared from bone marrow (BMDMs) of C57 mice also caused neuron loss, while macrophages from MFGE8 KO mice did not (Figure S2F; $p < 0.01$).

We noted several examples of IB4^{+} phagocytes in close proximity to cultured pFTAA^{+} neurons and some internalization of pFTAA^{+} aggregates (Figure 4C). To examine whether endogenous phagocytes were responsible for the loss of tau aggregate-containing neurons that occurs over 2–3 weeks, we eliminated the phagocytes by treatment (4 hr) with leucine methyl ester (LME) (Jebelli et al., 2015), followed by an overnight incubation to ensure death of phagocytes. We found that ~90% of the pFTAA^{+} neurons were maintained after phagocytes were eliminated from the cultures, whereas only ~70% of the neurons remained in untreated cultures after 14 days (Figure 4D; $p < 0.0001$). Because it was reported recently that living necroptotic cells also externalize PS (Zargarian et al., 2017), we tested whether the neurons were dying by necroptosis. In contrast to the abolition of neuron loss caused by LME, neuron loss was not prevented by the RIPK1 inhibitor necrostatin-1 (Degterev et al., 2008), indicating that neuron loss was not due to necroptosis (Figure S4). Thus, four types of phagocytes (BV2 cells, primary microglia, BMDMs, and resident phagocytes) are activated by pFTAA^{+} neurons that then phagocytose them, thereby causing neuronal loss.

Insoluble Tau Inclusions Are Transferred into Microglia after Phagocytosis

To further demonstrate phagocytosis of pFTAA^{+} neurons with tau inclusions by microglia, we pre-labeled BV2 cells with IB4-Alexa Fluor 594 before adding them to pFTAA pre-labeled neurons from 5-month-old P301S or C57 mice. Both cell types were washed extensively before co-culture to remove free dyes. After 4 days, BV2 cells were collected and sorted by fluorescence-activated cell sorting (FACS). Figure 4E shows that a minor but significant proportion of BV2 cells was double-labeled with pFTAA and IB4-594 in the population exposed to P301S-tau-derived DRG neurons (4.9%, gated fraction P6), but none was present in the P6-gated population exposed to C57-derived DRG neurons, indicating that the BV2 cells had engulfed pre-labeled tau inclusions. To confirm that the pFTAA^{+} material inside BV2 cells was insoluble tau, sorted cells were lysed in 5% SDS and filtered through a cellulose acetate membrane trap (Figure 4F). Tau from the P6-gated pFTAA/IB4^{+} population was retained on the filter, as detected by HT7, but no tau was retained from the C57 P6-gated population or the P4-gated single-labeled BV2 population. Sarkosyl-insoluble tau extracted from 5-month-old P301S mouse brain served as the positive control (Allen et al., 2002). These data indicate that pFTAA^{+} tau aggregates are transferred to BV2 cells during their co-culture with pFTAA^{+} DRG neurons, confirming that phagocytosis had occurred.

Many instances of pFTAA^{+} P301S-tau were also detected in microglia in brains from 5-month-old P301S mice. Figure 4G shows an example of a double-labeled pFTAA/HT7^{+} neuron with an intact nucleus inside an Iba1-stained microglial cell in the facial nucleus (FN). Engulfment of the whole neuron is evident in the confocal image (Figure 4G). Similar examples were present in the cortex from P301S mice (Figure S4).

Brains from 5-month-old P301S mice showed significant alterations in microglial morphology in the FN and other areas of the brain stem rich in tau aggregates, with marked increases in

OPEN
ACCESS
CellPress

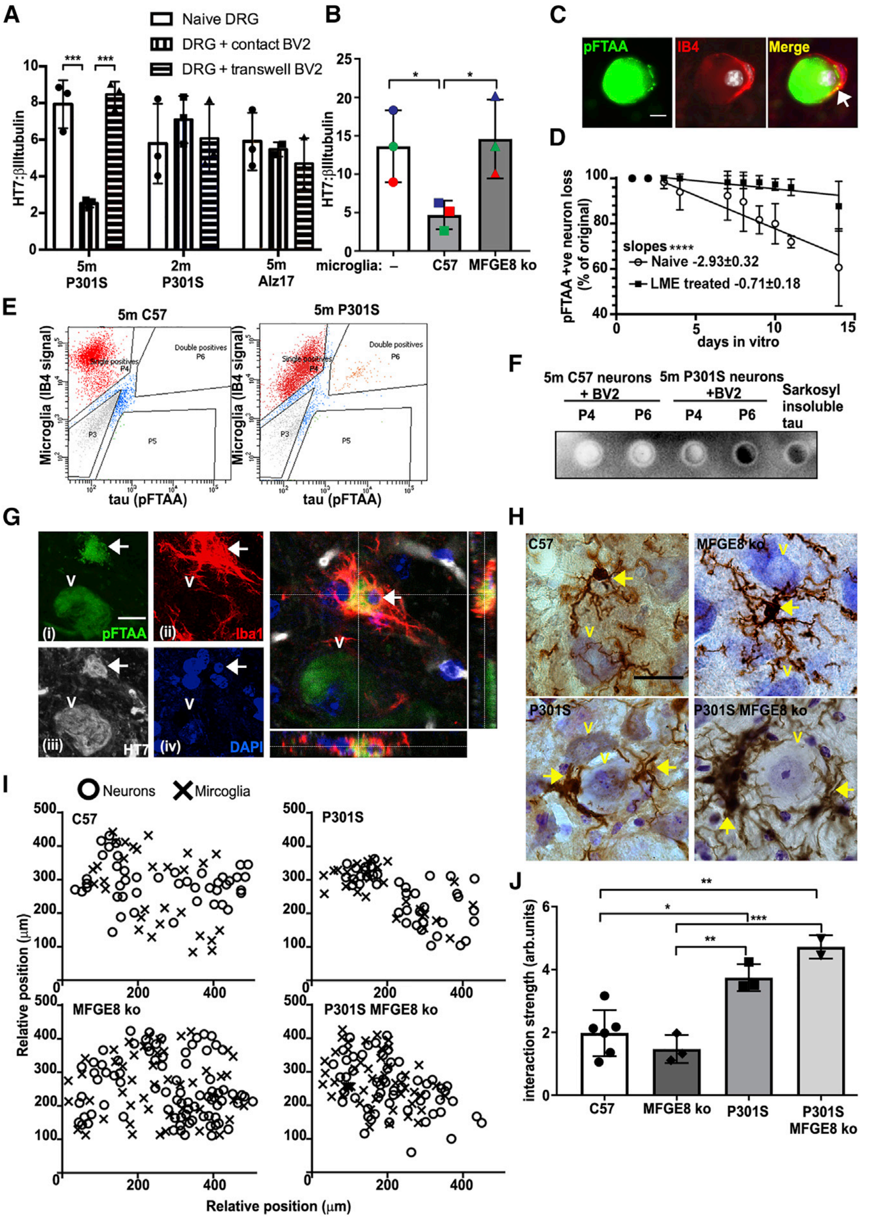

Figure 4. pFTAA/HT7$^+$ DRG Neurons Are Preferentially Removed by Phagocytosis

(A) pFTAA$^+$ DRG neurons from 5-month-old P301S mice are depleted after contact co-culture with BV2 cells for 4 days (***p < 0.001), but not if BV2 cells are in transwells. No loss of HT7$^+$ neurons when BV2 cells are contact co-cultured with neurons from 2-month-old P301S mice or 5-month-old Alz17 mice. Mean ± SD, n = 3 independent experiments, two-way ANOVA, Bonferroni corrected.

(B) pFTAA$^+$ DRG neurons from 5-month-old P301S mice are removed from cultures in contact with primary microglia from C57 mice for 4 days but not those from MFGE8 KO mice. Mean ± SD, n = 3 independent experiments, *p < 0.05, one-way ANOVA, Bonferroni corrected.

(C) Endogenous phagocytes mediate the slow removal of cultured pFTAA$^+$ neurons from 5-month-old P301S mice. Representative image of a pFTAA$^+$ neuron (green) surrounded by an IB4$^+$ phagocyte (red). Nuclei (DAPI) in white. Arrow marks pFTAA$^+$ inclusions inside the phagocyte.

(D) Removal of pFTAA$^+$ neurons over 14 days is halted by elimination of phagocytes using LME (50 mM). x axis, days *in vitro* beginning 1 day after phagocyte elimination. Each point shows mean ± SD, n = 3 independent experiments. Lines denote linear regressions depicting rates of neuronal loss; slopes, ****p < 0.0001.

(E) Insoluble pFTAA$^+$ tau is transferred from pFTAA$^+$ DRG neurons to BV2 cells in co-contact cultures. FACS sorting of IB4-594 pre-labeled BV2 cells collected 4 days after co-culture with pFTAA$^+$ neurons from 5-month-old P301S-tau or C57 mice. Note double-positive population (4.9%, P6) of BV2 cells present only in cultures from P301S mice.

(F) Insoluble tau in BV2 cells. Single IB4-labeled (P4) and double-labeled (P6) populations of BV2 cells shown in (E), extracted in 5% SDS and filtered through cellulose nitrate membrane, which traps insoluble tau. Sarkosyl-insoluble tau fibrils from 5-month-old P301S-tau brains are the positive control.

(G) Microglia in the facial nucleus of 5-month-old P301S-mice engulf pFTAA$^+$ neurons with tau. Total fluorescent staining of (i) pFTAA$^+$ neurons, (ii) Iba$^+$ microglia (red), (iii) human (HT7) P301S-tau (white), (iv) nuclei (DAPI, blue). Enlarged image: confocal z section shows the entire pFTAA$^+$ neuron encased inside a microglial cell. The neuronal nucleus (arrow) is also engulfed. Scale bar, 5 μm.

(H) Iba-1 staining of microglia in the FN from 5-month-old C57, 5-month-old P301S-tau, 5-month-old P301S-tau/MFGE8 KO with tau pathology, or MFGE8 KO mice. FN neurons are closely apposed by globoid-like microglia. Brown, Iba-1/DAB; blue, cresyl violet. Scale bar, 10 μm.

(I) Relative positions of motor neurons and microglia in representative examples of the FN. Microglia and neurons are more closely distributed in mice with P301S-tau pathology.

(J) Quantification of proximity; significant increases in interaction strength between P301S-tau$^+$ neurons and microglia in P301S-tau and P301S-tau/MFGE8 KO mice compared with C57 or MFGE8 KO controls. *p < 0.05, **p < 0.01, and ***p < 0.001, one-way ANOVA, Bonferroni corrected.

microglial proximity to neurons (Figure 4H), as previously noted (Bellucci et al., 2004). Interestingly, the neurons against which microglia were closest showed an altered morphology, although their nuclei retained a normal appearance. To establish that the proximity of tau aggregate-bearing neurons to microglia was induced by the presence of P301S-tau, we mapped the relative spatial distribution of neurons and microglia (Figures 4I and 4J). A clustered pattern of microglia intermingled with neurons was evident in brains of P301S mice compared with C57 or MFGE8 KO mice (Figure 4I). Quantification showed that there was a 2-fold increase in the interaction strength between microglia and neurons in P301S mice compared with C57 controls (Figure 4J). However, microglia were also closely associated with the neurons (Figure 4I, quantified in Figure 4J) in

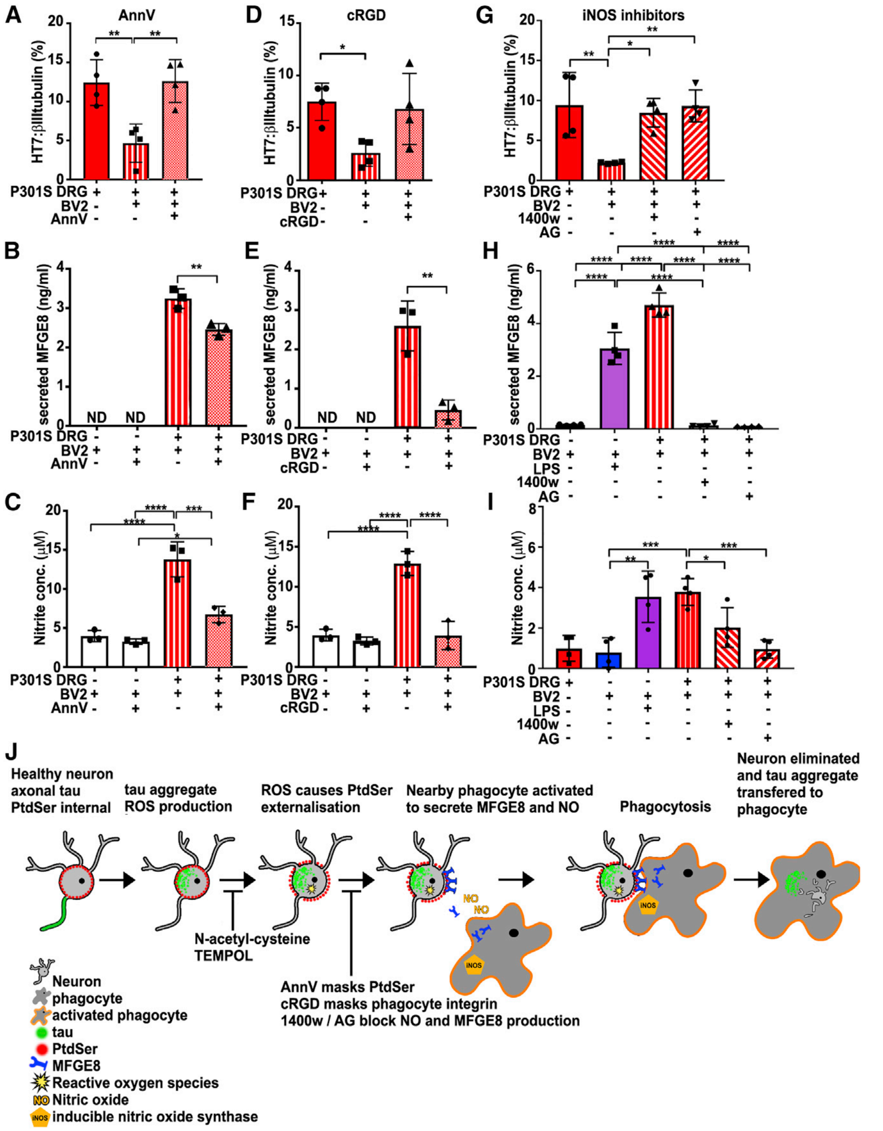

Figure 5. Blocking Phagocytosis Prevents the Loss of HT7⁺ Neurons by BV2 Cells

(A–C) Excess AnnV (100 nM) prevents loss of HT7$^+$ neurons (A) (**$p < 0.01$) and significantly reduces the amount of secreted MFGE8 (B) (**$p < 0.01$) and NO (C) (***$p < 0.001$). Mean ± SD, n = 4 independent experiments, one-way ANOVA, Bonferroni corrected.

(D–F) cRGD (5 μM) partially prevents the loss of HT7$^+$ neurons ($p = 0.09$) (D) and significantly reduces the amount of secreted MFGE8 (E) (**$p < 0.01$) and NO (F) (****$p < 0.0001$). Mean ± SD, n = 3 independent experiments, one-way ANOVA, Bonferroni corrected.

(G–I) The iNOS inhibitors 1400W and aminoguanidine (AG) inhibit the loss of HT7$^+$ neurons (G) (1400W, *$p < 0.05$; AG, **$p < 0.01$) and the production of MFGE8 (H) (1400W or AG, ****$p < 0.0001$) and NO (I) (1400W, *$p < 0.05$; AG, ***$p < 0.001$). LPS added as a positive control. Mean ± SD, n = 4 independent experiments, one-way ANOVA, Bonferroni corrected.

(J) Scheme summarizing the mechanism of death of tau aggregate-bearing neurons by phagocytosis: tau aggregate formation causing ROS-dependent PS exposure, activation of nearby phagocytes to produce NO and MFGE8, leading to engulfment of neurons and tau transfer into phagocytes. Blunt arrows indicate points of mechanism-related drug interception.

5-month-old double homozygous MFGE8 KO/P301S mice with tau aggregates. Thus, the change in location of microglia depended more on the state of neurons containing P301S-tau aggregates than on MFGE8 *per se* that instead seems necessary for phagocytosis.

Inhibiting Phagocytosis Rescues Living Neurons

To show further evidence that neurons with tau inclusions are eaten alive in an MFGE8-dependent manner, we co-cultured neurons from 5-month-old P301S mice with BV2 cells and added either excess AnnV to mask neuronal PS (Krahling et al., 1999; Neher et al., 2011) or the cyclo peptide cRGD*f*V (cRGD), to mask MFGE8 RGD binding to the αv vitronectin receptor subunit (Asano et al., 2004). AnnV significantly reduced the loss of tau$^+$ (HT7$^+$) neurons from the cultures ($p < 0.01$), while the cRGD peptide showed a trend toward protection (Figures 5A and 5D). Interestingly, both treatments reduced MFGE8 and NO secretion in the co-cultures (Figures 5B, $p < 0.01$ and 5C, $p < 0.001$). Reduced phagocytosis was not due to *a priori* inactivation of BV2 microglia by AnnV or the cRGD peptide, as neither compound reduced MFGE8 expression or MFGE8 and NO release when added to LPS-activated cultures of BV2 cells (Figures S5A–S5C). Hence, phagocytosis induced by the DRG neurons with tau inclusions requires MFGE8 and NO production in BV2 cells, which, when abrogated, leads to the rescue of living neurons.

Because AnnV and cRGD treatments inhibited both MFGE8 and NO production, we investigated whether direct inhibition of NO production using inducible NO synthase (iNOS) inhibitors would also abrogate phagocytosis. The results show that N-(3-(aminomethyl)benzyl)acetamidine (1400W) or aminoguanidine (AG) significantly reduced the production of NO from BV2 cells induced by neurons from 5-month-old P301S mice (Figure 4I; 1400W, $p < 0.05$; AG, $p < 0.001$) and that both compounds inhibited the loss of tau$^+$ neurons (Figure 4G; 1400W, $p < 0.05$; AG, $p < 0.01$). Moreover, the iNOS inhibitors also abrogated the production of MFGE8 (Figure 4H; $p < 0.001$), further demonstrating the reciprocity between NO signaling and MFGE8 production. Thus, NO induced in phagocytes by neurons with tau inclusions is an essential component of the phagocytic process.

DISCUSSION

The presence of tau filaments is correlated with neurodegeneration in familial and sporadic tauopathies, but the mechanisms by which assembled tau may cause neuronal cell death remain elusive (Spillantini and Goedert, 2013). This study implicates neurons with intracellular filamentous tau aggregates in instigating a type of cell death whereby living neurons under stress expose the "eat me" signal PS and activate phagocytic cells that recognize, engulf, and dispose them while still alive. Our study adds neurons with tau inclusions to a growing list of cells that are phagocytosed while still alive as a homeostatic mechanism to regulate cell numbers and remove potentially unfit cells (Merino et al., 2015).

It has been suggested that neuronal cell death in neurodegenerative diseases is the consequence of a breakdown of cooperative processes between neurons and other cells, not least microglia (De Strooper and Karran, 2016). Microglia are the resident immune cells of the brain that sometimes interact beneficially with surrounding cells and sometimes have deleterious effects, especially during inflammation (Aguzzi et al., 2013; Boche et al., 2013). By instigating the exposure of PS, intracellular P301S-tau aggregates cause microglia to release opsonins, which facilitate engulfment, and NO that can perpetuate an inflammatory state. In contrast to its exposure in apoptosis, here PS could be re-internalized, emphasizing the fact that the neurons are alive, and it is phagocytosis that kills them.

Interest is increasing in inflammation as contributing to neurodegeneration (Bolós et al., 2017). A role for innate immune factors has long been acknowledged in Alzheimer's disease (AD) (Eikelenboom and Stam, 1982) and some tauopathies (Singhrao et al., 1996), but how neuroinflammation is instigated in diseases with intracellular protein aggregates in unclear. Here, neurons with filamentous tau aggregates led to microglial activation through close contact, establishing a causal relationship between intracellular aggregation and an inflammatory response. The signaling pathway starts in the neurons by tau aggregate-mediated ROS production, causing neuronal PS exposure, followed by NO production in the phagocytes that regulates release of MFGE8 necessary for phagocytosis (see Figure 5 for summary scheme). We further extend the model in which NO is postulated to promote release of MFGE8 (Hornik et al., 2016) by proposing that MFGE8 released as a result of iNOS/NO stimulation feeds back onto the $\alpha v\beta 3$ receptor to amplify iNOS/NO output. Indeed, $\alpha v\beta 3$ has been implicated in the activation of astrocytes, which also produce MFGE8 in the brain (Lagos-Cabré et al., 2017). It is interesting that lack of MFGE8 did not prevent the proximity of microglia to neurons in the facila nucleus of 5-month-old double crossed MFGE8 KO/P301S-tau$^{+/+}$ mice. However, many other pairs of signals are expressed on target cells and phagocytes that might facilitate proximity, even though MFGE8 is still required for phagocytosis (Neher et al., 2013; Rothlin et al., 2015). Which of these signals predominate under each disease condition remains to be explored.

Phagocytosis of alive tau inclusion-bearing neurons may have implications for potential therapies. We found increased expression of MFGE8 in the frontal cortex of FTD cases with *MAPT* mutations (P301L and FTDP+3) or sporadic Pick's disease but not in the cerebellum of P301L cases, showing that the expression level of MFGE8 depends on the presence of tau aggregates. Preservation of neurons, albeit with tau inclusions, preserves a working network and provides a substrate for potential therapies, which by altering aggregation and toxicity may well be able to prevent neuronal loss.

STAR★METHODS

Detailed methods are provided in the online version of this paper and include the following:

- KEY RESOURCES TABLE
- CONTACT FOR REAGENT AND RESOURCE SHARING
- EXPERIMENTAL MODEL AND SUBJECT DETAILS
  - Animals
  - Human tissue
  - Cultures
- METHOD DETAILS
  - Live cell labeling
  - LME treatment
  - Anti-oxidant and arsenite treatments
  - Immunocytochemistry
  - Immunohistochemistry
  - Immunoblotting
  - Fluorescence-activated cell sorting (FACS)
  - NO assay
  - MFGE8 ELISA
  - Interaction analysis
- QUANTIFICATION AND STATISTICAL ANALYSIS

SUPPLEMENTAL INFORMATION

Supplemental Information includes five figures and can be found with this article online at https://doi.org/10.1016/j.celrep.2018.07.072.

ACKNOWLEDGMENTS

We thank K. Peter Nilsson (Linköping University, Sweden) for providing pFTAA, Clotilde Théry (Institut Curie, PSL Research University, France) for MFGE8 KO mice, the Cambridge Brain Bank for providing human tissues, Greg Strachan (Cambridge Metabolic Research Laboratory Imaging Core (funded by a Wellcome Trust Strategic Award [100574/Z/12/Z])) for help with microscopy and image analysis, Emma Carlson for preparation of brain sections, and Emma and Heather Lloyd for animal husbandry. Guy Brown (University of Cambridge) and Jonas Neher (University of Tübingen) provided valuable advice and discussion. This work was supported by grant NC/L000741/1 from the National Council for the 3Rs (NC3Rs) (M.G.S. and A.M.T.), grant PG2011-20 from Alzheimer's Research UK (ARUK) (M.G.S.), an ARUK Junior Fellowship (J.B.), grant P30-AG010133 from the NIH (B.G.), and grant MC_U105184291 from the UK Medical Research Council (M.G.). The Cambridge Brain Bank is supported by the National Institute for Health Research (NIHR) Cambridge Biomedical Research Centre.

AUTHOR CONTRIBUTIONS

Conceptualization, J.B., A.M.T., and M.G.S.; Methodology, J.B. and A.M.T.; Investigation: J.B., A.M.T., and B.G.; Resources, M.G. and B.G; Writing – Original Draft: J.B. and A.M.T.; Writing – Review & Editing, J.B., A.M.T., B.G., M.G., and M.G.S.; Funding Acquisition, M.G.S., A.M.T., B.G., M.G., and J.B.; Supervision, A.M.T, M.G.S., and M.G.

DECLARATION OF INTERESTS

The authors declare no competing interests.

Received: December 4, 2017
Revised: May 23, 2018
Accepted: July 21, 2018
Published: August 21, 2018

REFERENCES

Aguzzi, A., Barres, B.A., and Bennett, M.L. (2013). Microglia: scapegoat, saboteur, or something else? Science *339*, 156–161.

Allen, B., Ingram, E., Takao, M., Smith, M.J., Jakes, R., Virdee, K., Yoshida, H., Holzer, M., Craxton, M., Emson, P.C., et al. (2002). Abundant tau filaments and nonapoptotic neurodegeneration in transgenic mice expressing human P301S tau protein. J. Neurosci. *22*, 9340–9351.

Asano, K., Miwa, M., Miwa, K., Hanayama, R., Nagase, H., Nagata, S., and Tanaka, M. (2004). Masking of phosphatidylserine inhibits apoptotic cell engulfment and induces autoantibody production in mice. J. Exp. Med. *200*, 459–467.

Bellucci, A., Westwood, A.J., Ingram, E., Casamenti, F., Goedert, M., and Spillantini, M.G. (2004). Induction of inflammatory mediators and microglial activation in mice transgenic for mutant human P301S tau protein. Am. J. Pathol. *165*, 1643–1652.

Bellucci, A., Bugiani, O., Ghetti, B., and Spillantini, M.G. (2011). Presence of reactive microglia and neuroinflammatory mediators in a case of frontotemporal dementia with P301S mutation. Neurodegener. Dis. *8*, 221–229.

Boche, D., Perry, V.H., and Nicoll, J.A. (2013). Review: activation patterns of microglia and their identification in the human brain. Neuropathol. Appl. Neurobiol. *39*, 3–18.

Boddaert, J., Kinugawa, K., Lambert, J.C., Boukhtouche, F., Zoll, J., Merval, R., Blanc-Brude, O., Mann, D., Berr, C., Vilar, J., et al. (2007). Evidence of a role for lactadherin in Alzheimer's disease. Am. J. Pathol. *170*, 921–929.

Bolós, M., Llorens-Martín, M., Jurado-Arjona, J., Hernández, F., Rábano, A., and Avila, J. (2016). Direct evidence of internalization of tau by microglia in vitro and in vivo. J. Alzheimers Dis. *50*, 77–87.

Bolós, M., Perea, J.R., and Avila, J. (2017). Alzheimer's disease as an inflammatory disease. Biomol. Concepts *8*, 37–43.

Brelstaff, J., Ossola, B., Neher, J.J., Klingstedt, T., Nilsson, K.P., Goedert, M., Spillantini, M.G., and Tolkovsky, A.M. (2015a). The fluorescent pentameric oligothiophene pFTAA identifies filamentous tau in live neurons cultured from adult P301S tau mice. Front. Neurosci. *9*, 184.

Brelstaff, J., Spillantini, M.G., and Tolkovsky, A.M. (2015b). pFTAA: a high affinity oligothiophene probe that detects filamentous tau in vivo and in cultured neurons. Neural Regen. Res. *10*, 1746–1747.

Brown, G.C., and Neher, J.J. (2014). Microglial phagocytosis of live neurons. Nat. Rev. Neurosci. *15*, 209–216.

Brown, G.C., Vilalta, A., and Fricker, M. (2015). Phagoptosis—cell death by phagocytosis—plays central roles in physiology, host defense and pathology. Curr. Mol. Med. *15*, 842–851.

Bugiani, O., Murrell, J.R., Giaccone, G., Hasegawa, M., Ghigo, G., Tabaton, M., Morbin, M., Primavera, A., Carella, F., Solaro, C., et al. (1999). Frontotemporal dementia and corticobasal degeneration in a family with a P301S mutation in tau. J. Neuropathol. Exp. Neurol. *58*, 667–677.

De Strooper, B., and Karran, E. (2016). The cellular phase of Alzheimer's disease. Cell *164*, 603–615.

Degterev, A., Hitomi, J., Germscheid, M., Ch'en, I.L., Korkina, O., Teng, X., Abbott, D., Cuny, G.D., Yuan, C., Wagner, G., et al. (2008). Identification of RIP1 kinase as a specific cellular target of necrostatins. Nat. Chem. Biol. *4*, 313–321.

Delobel, P., Lavenir, I., Fraser, G., Ingram, E., Holzer, M., Ghetti, B., Spillantini, M.G., Crowther, R.A., and Goedert, M. (2008). Analysis of tau phosphorylation and truncation in a mouse model of human tauopathy. Am. J. Pathol. *172*, 123–131.

Eikelenboom, P., and Stam, F.C. (1982). Immunoglobulins and complement factors in senile plaques. An immunoperoxidase study. Acta Neuropathol. *57*, 239–242.

Hanayama, R., Tanaka, M., Miwa, K., Shinohara, A., Iwamatsu, A., and Nagata, S. (2002). Identification of a factor that links apoptotic cells to phagocytes. Nature *417*, 182–187.

Hornik, T.C., Vilalta, A., and Brown, G.C. (2016). Activated microglia cause reversible apoptosis of pheochromocytoma cells, inducing their cell death by phagocytosis. J. Cell Sci. *129*, 65–79.

Hutton, M., Lendon, C.L., Rizzu, P., Baker, M., Froelich, S., Houlden, H., Pickering-Brown, S., Chakraverty, S., Isaacs, A., Grover, A., et al. (1998). Association of missense and 5′-splice-site mutations in tau with the inherited dementia FTDP-17. Nature *393*, 702–705.

Jebelli, J., Piers, T., and Pocock, J. (2015). Selective depletion of microglia from cerebellar granule cell cultures using L-leucine methyl ester. J. Vis. Exp. (*101*), e52983.

Johnsen, H.L., and Horvitz, H.R. (2016). Both the apoptotic suicide pathway and phagocytosis are required for a programmed cell death in Caenorhabditis elegans. BMC Biol. *14*, 39.

Kawasaki, H., Murayama, S., Tomonaga, M., Izumiyama, N., and Shimada, H. (1987). Neurofibrillary tangles in human upper cervical ganglia. Morphological study with immunohistochemistry and electron microscopy. Acta Neuropathol. *75*, 156–159.

Krahling, S., Callahan, M.K., Williamson, P., and Schlegel, R.A. (1999). Exposure of phosphatidylserine is a general feature in the phagocytosis of apoptotic lymphocytes by macrophages. Cell Death Differ. *6*, 183–189.

Kranich, J., Krautler, N.J., Falsig, J., Ballmer, B., Li, S., Hutter, G., Schwarz, P., Moos, R., Julius, C., Miele, G., and Aguzzi, A. (2010). Engulfment of cerebral apoptotic bodies controls the course of prion disease in a mouse strain-dependent manner. J. Exp. Med. *207*, 2271–2281.

Lagos-Cabré, R., Alvarez, A., Kong, M., Burgos-Bravo, F., Cárdenas, A., Rojas-Mancilla, E., Pérez-Nuñez, R., Herrera-Molina, R., Rojas, F., Schneider, P., et al. (2017). $\alpha_V\beta_3$ Integrin regulates astrocyte reactivity. J. Neuroinflammation *14*, 194.

Maphis, N., Xu, G., Kokiko-Cochran, O.N., Jiang, S., Cardona, A., Ransohoff, R.M., Lamb, B.T., and Bhaskar, K. (2015). Reactive microglia drive tau pathology and contribute to the spreading of pathological tau in the brain. Brain *138*, 1738–1755.

Mellone, M., Kestoras, D., Andrews, M.R., Dassie, E., Crowther, R.A., Stokin, G.B., Tinsley, J., Horne, G., Goedert, M., Tolkovsky, A.M., and Spillantini, M.G. (2013). Tau pathology is present in vivo and develops in vitro in sensory neurons from human P301S tau transgenic mice: a system for screening drugs against tauopathies. J. Neurosci. *33*, 18175–18189.

Merino, M.M., Rhiner, C., Lopez-Gay, J.M., Buechel, D., Hauert, B., and Moreno, E. (2015). Elimination of unfit cells maintains tissue health and prolongs lifespan. Cell *160*, 461–476.

Mirra, S.S., Murrell, J.R., Gearing, M., Spillantini, M.G., Goedert, M., Crowther, R.A., Levey, A.I., Jones, R., Green, J., Shoffner, J.M., et al. (1999). Tau pathology in a family with dementia and a P301L mutation in tau. J. Neuropathol. Exp. Neurol. *58*, 335–345.

Nagata, S., Suzuki, J., Segawa, K., and Fujii, T. (2016). Exposure of phosphatidylserine on the cell surface. Cell Death Differ. *23*, 952–961.

Neher, J.J., Neniskyte, U., Zhao, J.W., Bal-Price, A., Tolkovsky, A.M., and Brown, G.C. (2011). Inhibition of microglial phagocytosis is sufficient to prevent inflammatory neuronal death. J. Immunol. *186*, 4973–4983.

Neher, J.J., Emmrich, J.V., Fricker, M., Mander, P.K., Théry, C., and Brown, G.C. (2013). Phagocytosis executes delayed neuronal death after focal brain ischemia. Proc. Natl. Acad. Sci. U S A *110*, E4098–E4107.

Nishimura, M., Namba, Y., Ikeda, K., Akiguchi, I., and Oda, M. (1993). Neurofibrillary tangles in the neurons of spinal dorsal root ganglia of patients with progressive supranuclear palsy. Acta Neuropathol. *85*, 453–457.

Probst, A., Götz, J., Wiederhold, K.H., Tolnay, M., Mistl, C., Jaton, A.L., Hong, M., Ishihara, T., Lee, V.M., Trojanowski, J.Q., et al. (2000). Axonopathy and amyotrophy in mice transgenic for human four-repeat tau protein. Acta Neuropathol. *99*, 469–481.

Rothlin, C.V., Carrera-Silva, E.A., Bosurgi, L., and Ghosh, S. (2015). TAM receptor signaling in immune homeostasis. Annu. Rev. Immunol. *33*, 355–391.

Salter, M.W., and Stevens, B. (2017). Microglia emerge as central players in brain disease. Nat. Med. *23*, 1018–1027.

Sarkar, A., Sengupta, D., Mandal, S., Sen, G., Dutta Chowdhury, K., and Chandra Sadhukhan, G. (2015). Treatment with garlic restores membrane thiol content and ameliorates lead induced early death of erythrocytes in mice. Environ. Toxicol. *30*, 396–410.

Sawada, M., Suzumura, A., Yamamoto, H., and Marunouchi, T. (1990). Activation and proliferation of the isolated microglia by colony stimulating factor-1 and possible involvement of protein kinase C. Brain Res. *509*, 119–124.

Schreiber, R., Ousingsawat, J., Wanitchakool, P., Sirianant, L., Benedetto, R., Reiss, K., and Kunzelmann, K. (2018). Regulation of TMEM16A/ANO1 and TMEM16F/ANO6 ion currents and phospholipid scrambling by Ca^{2+} and plasma membrane lipid. J. Physiol. *596*, 217–229.

Silvestre, J.S., Théry, C., Hamard, G., Boddaert, J., Aguilar, B., Delcayre, A., Houbron, C., Tamarat, R., Blanc-Brude, O., Heeneman, S., et al. (2005). Lactadherin promotes VEGF-dependent neovascularization. Nat. Med. *11*, 499–506.

Singhrao, S.K., Neal, J.W., Gasque, P., Morgan, B.P., and Newman, G.R. (1996). Role of complement in the aetiology of Pick's disease? J. Neuropathol. Exp. Neurol. *55*, 578–593.

Spangenberg, E.E., and Green, K.N. (2017). Inflammation in Alzheimer's disease: Lessons learned from microglia-depletion models. Brain Behav. Immun. *61*, 1–11.

Spillantini, M.G., and Goedert, M. (2013). Tau pathology and neurodegeneration. Lancet Neurol. *12*, 609–622.

Spillantini, M.G., Goedert, M., Crowther, R.A., Murrell, J.R., Farlow, M.R., and Ghetti, B. (1997). Familial multiple system tauopathy with presenile dementia: a disease with abundant neuronal and glial tau filaments. Proc. Natl. Acad. Sci. U S A *94*, 4113–4118.

Tan, S., Schubert, D., and Maher, P. (2001). Oxytosis: a novel form of programmed cell death. Curr. Top. Med. Chem. *1*, 497–506.

Trouplin, V., Boucherit, N., Gorvel, L., Conti, F., Mottola, G., and Ghigo, E. (2013). Bone marrow-derived macrophage production. J. Vis. Exp. (*81*), e50966.

Tyurin, V.A., Balasubramanian, K., Winnica, D., Tyurina, Y.Y., Vikulina, A.S., He, R.R., Kapralov, A.A., Macphee, C.H., and Kagan, V.E. (2014). Oxidatively modified phosphatidylserines on the surface of apoptotic cells are essential phagocytic 'eat-me' signals: cleavage and inhibition of phagocytosis by Lp-PLA2. Cell Death Differ. *21*, 825–835.

Yang, W.S., and Stockwell, B.R. (2016). Ferroptosis: death by lipid peroxidation. Trends Cell Biol. *26*, 165–176.

Zargarian, S., Shlomovitz, I., Erlich, Z., Hourizadeh, A., Ofir-Birin, Y., Croker, B.A., Regev-Rudzki, N., Edry-Botzer, L., and Gerlic, M. (2017). Phosphatidylserine externalization, "necroptotic bodies" release, and phagocytosis during necroptosis. PLoS Biol. *15*, e2002711.

STAR★METHODS

KEY RESOURCES TABLE

REAGENT or RESOURCE	SOURCE	IDENTIFIER
Antibodies		
Human tau (HT7) (1:500)	Thermo Fisher Scientific	Cat# MN1000; RRID:AB_2314654
β-III-tubulin (1:1000)	Covance Research Products	Cat# PRB-435P-100; RRID:AB_291637
Mouse MFGE8 (tissue/blot, 1:1000)	R&D Systems	Cat# AF2805; RRID:AB_2281868
Mouse MFGE8 (ELISA, 1:500)	R&D Systems	Cat# MAB2805; RRID:AB_2297564
Human MFGE8	R&D Systems	Cat# AF2767; RRID:AB_10889829
Iba-1	WAKO	Cat# 016-20001; RRID:AB_839506
Active Caspase 3 Asp175	Cell Signaling	Cat# 9661; RRID:AB_868672
AlexaFluor 488/568/647/350 conjugated secondary various species	Thermo Fisher Scientific	N/A
Biotinylated secondary various species	Vector	N/A
HRP-conjugated secondary various species	GE Healthcare	N/A
Biological Samples		
Human brain tissue	B Ghetti, IADC, Indiana University<br>Cambridge Brain Bank	https://medicine.iu.edu/research/centers-institutes/alzheimers/servicecores/neuropathology/<br>https://www.cuh.nhs.uk/for-public/cambridge-brain-bank
Chemicals, Peptides, and Recombinant Proteins		
pFTAA (3 μM)	Gift from K Peter Nilsson	https://doi.org/10.1021/bi100922r
N-acetyl cysteine (NAC) (5 mM)	Sigma-Aldrich	Cat# A7250
4-Hydroxy-TEMPO (TEMPOL) (5 mM)	Sigma-Aldrich	Cat# 176141
Cyclo(RGD*f*V) (cRGD) peptide (5 μM)	BACHEM	Cat# H2574
Recombinant AnnV (100 nM)	Immunotools	Cat# 31490010
AlexaFluor 647-conjugated AnnV (5% v/v)	Thermo Fisher Scientific	Cat# A23204
Recombinant mouse MFGE8	R&D Systems	Cat# 2805-MF-050/CF
Lipopolysaccharide (LPS) (100 nM)	Sigma	Cat# L4391
AlexaFluor-594-IB4 (1:200)	Thermo Fisher Scientific	Cat# I21413,
Necrostatin-1 (Nec-1) (10 μM),	Sigma-Aldrich	Cat# N9037
Nec-1i (inactive analog) (10 μM)	Calbiochem	Cat# 480066
1400W (25 μM)	Alexa	Cat# 270-073-M005
aminoguanidine (200 μM)	Sigma-Aldrich	Cat# 396494
sodium meta arsenite (0.5 mM)	Sigma-Aldrich	Cat# S71287
Boc-Asp(Omethyl)FMK (BAF) (50 μM)	MP Biomedicals	Cat# 03FK011
Staurosporine (250 nM)	Sigma-Aldrich	Cat# S4400
Calcein-AM (10 μM)	Invitrogen Thermo Fisher	Cat# C1429
Dihydroethidium (2.5 μM)	Invitrogen Thermo Fisher	Cat# D1168
L-Leucine methyl ester (50 mM)	Sigma-Aldrich	Cat# L1002-
Critical Commercial Assays		
VECTASTAIN Elite ABC-Peroxidase Kit	Vector Laboratories	Cat# PK-6100
Mouse MFG-E8 Quantikine ELISA Kit	R&D Systems	Cat# MFGE80
Griess NO kit	Abcam	Cat# ab65328
DAB Peroxidase kit	Vector Laboratories	Cat# SK-4100

(*Continued on next page*)

Continued

REAGENT or RESOURCE	SOURCE	IDENTIFIER
Experimental Models: Cell Lines		
BV2 cells	Gift from Miguel Burgillios	N/A
L929 cells	Gift from Stefano Pluchino's lab	N/A
Experimental Models: Organisms/Strains		
Mouse MFGE8 KO/C57BL/6J Bkg	Gift from Clothilde Théry	Silvestre et al., 2005
Mouse P301S-tau 0N4R on C57BL/6J or C57BL/6S Bkg	M Goedert	Allen et al., 2002
Mouse MFGE8KOxP301S-tau+/+ on C57BL6/J Bkg	M Goedert	This paper
Mouse Alz17 (2N4R tau) on C57BL/6S Bkg	M Goedert	Probst et al., 2000
Software and Algorithms		
MosaicIA	N/A	https://imagej.net/MOSAICsuite
ImageJ	N/A	https://imagej.net/ImageJ
Graph Pad Prism version 7	https://www.graphpad.com/scientific-software/prism/	N/A

CONTACT FOR REAGENT AND RESOURCE SHARING

Further information and requests for resources and reagents should be directed to and will be fulfilled by the Lead Contact, Maria Grazia Spillantini (mgs11@cam.ac.uk).

EXPERIMENTAL MODEL AND SUBJECT DETAILS

Animals

Mouse and human study protocols were approved by the Local Review and Ethics Committees (LREC) of University of Cambridge and the Indiana University Institutional Review Board. Homozygous mice transgenic for human mutant 0N4R P301S-tau or wild-type human 2N4R tau (Alz17) (Probst et al., 2000), and C57BL/6:OlaHsd (Harlan) background-matched control mice were maintained as described previously (Mellone et al., 2013). MFGE8 KO mice express a mutant MFGE8 lacking one of the two obligatory vitronectin receptor binding sites and a transmembrane sequence that tethers the protein in the ER membrane thereby abrogating its secretion (Silvestre et al., 2005). MFGE8 KO mice were crossed with P301S-taumice in a C57BL/6:Jax background to produce double homozygous MFGE8 KO/ P301S-tau+/+ mice. Both female and male mice were used and each individual was from a different litter. Mice were housed in groups in individually ventilated cages, adding a cardboard roll and nesting material for enrichment. Mice were kept under a 12 h light/dark cycle, with food and water available *ad libitum*. Mash food was placed in the cage when the onset of pathology was evident, around 4 month of age. Our research was performed under the Animals (Scientific Procedures) Act 1986, Amendment Regulations 2012, following an ethical review by the University of Cambridge Animal Welfare and Ethical Review Body (AWERB).

Human tissue

Human brain tissue was obtained from The Alzheimer Disease Center, Indiana University School of Medicine and the Cambridge Brain Bank. Grey matter (0.2 g) was mechanically homogenized in RIPA buffer containing 2.5% SDS with phosphatase and protease inhibitors at a 1:2 (w/v) ratio. Lysate was clarified at 20,000 xg for 30 min and protein assayed.

Patient details are as follows:

Abbreviations: FTDP-17T, frontotemporal dementia and Parkinsonism linked to chromosome 17, MSTD, Multiple system tauopathy with presenile dementia, FTLD, frontotemporal lobar degeneration

Sample order (as on blot)	Disease or mutation	Gender	Age at death	Neuropathology
1	Healthy	F	56	Moderate cerebral atherosclerosis
2	Healthy	M	65	Metastatic adenocarcinoma
3	Healthy	F	66	Cerebral atrophy
4 and 7	P301L	M	59	FTDP-17T
5 and 8	P301L	F	55	FTDP-17T

(*Continued on next page*)

Continued

Sample order (as on blot)	Disease or mutation	Gender	Age at death	Neuropathology
6 and 9	P301L	F	62	FTDP-17T
10	MAPT+3	M	64	FTDP-17T (MSTD)
11	MAPT+3	F	58	FTDP-17T (MSTD)
12	MAPT+3	F	61	FTDP-17T (MSTD)
13	Pick's Disease	F	76	Tau^+ Pick bodies
14	Pick's Disease	M	75	Tau^+ Pick bodies
15	Pick's Disease	F	61	Tau^+ Pick bodies
16 and 19	C9ORF72	F	42	FTLD
17 and 20	C9ORF72	M	69	Neurodegenerative disease
18 and 21	C9ORF72	F	65	FTLD with TDP-43 type B

Cultures

DRG neurons were cultured in DMEM-based growth medium lacking anti-oxidants as described previously (Brelstaff et al., 2015a). Primary microglia were prepared from cortices of newborn C57 mice or MFGE8 KO mice and expanded in medium containing 40% DMEM with 10% heat inactivated FBS and 60% L929 cell conditioned medium (Sawada et al., 1990). BMDM cells were prepared from bone marrows of long limbs of 3-4 week-old mice (Trouplin et al., 2013). BV2 cells were expanded from an original stock obtained from ATCC. For co-culture, 200,000 BV2 cells or primary microglia or BMDM per culture (a ratio of about 20:1) were added either directly to the medium or physically separated using a 0.4 μm pore transwell insert (Costar). Co-cultures were maintained in DMEM, 1% PSF, 5% heat inactivated FBS, and 2 mM GlutaMax (GIBCO) for 4 days before analysis.

METHOD DETAILS

Live cell labeling

For live labeling with AnnV, neurons were cultured for 7 days, washed, and labeled with 3 μM pFTAA and 0.1 μg/ml PI or DAPI (Brelstaff et al., 2015a). Medium was exchanged for HBSS containing 2.5 mM $CaCl_2$ and 0.5% (v/v) Alexa Fluor®-647-conjugated AnnV for 15 min at RT in the dark, then returned to growth medium and imaged on a Leica DMI 4000B microscope using a Leica DFC3000 G camera and the Leica application suite 4.0.0.11706.

LME treatment

Following 4 h of incubation with LME (50 mM) in normal growth medium, LME was removed by 3 washes with PBS and cultures were returned into growth medium overnight to ensure the death of the endogenous phagocytes. The next day, cultures were stained with pFTAA, returned to growth medium and counted. The same fields of neurons were imaged over 14 days.

Anti-oxidant and arsenite treatments

NAC or TEMPOL (5 mM final concentration) were added in growth medium for 2 days after which cells were washed and labeled with AnnV-647. Arsenite (0.5 mM) was added for 15 min before washing and labeling with AnnV-647.

Immunocytochemistry

Dissociated DRG neurons were fixed either in 100% ethanol or 4% PFA for 20 min and permeabilised in PBS containing 0.3% triton x-100 (PBST). Antibodies were applied in PBST as described previously (Brelstaff et al., 2015a). Fluorescent images were captured on a Leica DMI 4000B microscope as described above. Images were analyzed using ImageJ (Rasband, W.S., ImageJ, U.S. National Institutes of Health, Bethesda, Maryland, USA, http://imagej.nih.gov/ij/,1997–2014). Confocal images were taken on a Leica SP8 TCS microscope using LAS X version 2.0.1.14392 software.

Immunohistochemistry

Fresh mouse brains were immediately fixed in 4% PFA for 24-48 h and sunk in PBS containing 30% sucrose. Coronal sections (25 μm) were cut on a freezing microtome (Bright Instruments). Floating sections were permeablised in PBST and stained in PBST with primary antibodies at 4°C, followed by the appropriate biotin-conjugated secondary antibodies. Staining was revealed using the VECTASTAIN Elite ABC HRP Kit followed by the DAB kit (Vectorlabs). Sections were counter stained in Cresyl Violet and mounted in DPX.

Immunoblotting

Cells were lysed in 1% NP40 lysis buffer with protease inhibitor cocktail (Roche). Equal protein amounts were loaded in LDS sample buffer (NuPAGE, ThermoFisher) onto 4%–12% gradient SDS-PAGE gels, and run with NuPAGE antioxidant (ThermoFisher). After blotting onto 0.2 μm pore PVDF membranes (Merck), nonspecific background was blocked in 5% w/v skimmed milk (Tesco) or 10% BSA in PBS containing 0.1% Tween-20 for 1 h and incubated with primary antibody overnight at 4°C, followed by the appropriate HRP-labeled secondary antibody. Blots were developed with ECL Dura (GE Healthcare). For the dot blot, FACS-sorted cells were pelleted and lysed in lysis buffer. DNA was digested by the addition of 50 μg/ml DNase-I (Sigma) in 50 mM Tris pH 8 with 10 mM $MgCl_2$ for 1 h at 37°C. Samples were incubated in 5% SDS in dH_2O for 10 min at room temperature and the entire mixture was filtered through a cellulose acetate membrane under vacuum. Membranes were washed in 5% SDS under vacuum, blocked in 5% skimmed milk in PBS for 30 min, then incubated with primary antibody (HT7) overnight at 4°C, and developed using the method described above. A sarkosyl-insoluble tau preparation from a 5 m P301S-tau mouse brain (Allen et al., 2002) and was used as a positive control.

Fluorescence-activated cell sorting (FACS)

Live neurons pooled from three 5 m P301S-tau or C57 mice per sample were cultured for 7 DIV, labeled with 3 μM pFTAA for 30 min at room temperature and washed. BV2 microglia were pre-labeled with IB4-594 for 15 min and washed before addition to DRG neuron cultures. After 4 days, BV2 cells were mechanically dislodged from DRG co-cultures, strained through a 40 μm pore filter (BD Falcon), and sorted on a BD FACSAriaFusion Cell Sorter (Cambridge NIHR BRC Cell Phenotyping Hub). Voltage range was set against unlabelled BV2 microglia and those singly labeled with IB4-594. Appropriate gates to exclude doublets and cellular debris were set and single or double positive populations were collected.

NO assay

Conditioned medium was deproteinised by centrifugation through a 10 KDa cutoff filter (Amicon UFC501024). NO was measured using a colorimetric Griess NO kit (ab65328 Abcam). Absorbance (540 nm) was measured on an InfiniteM200Pro Tecan plate reader.

MFGE8 ELISA

Conditioned medium was concentrated through a 10 KDa cutoff filter and diluted back to its original volume with PBS. MFGE8 was measured using a colorimetric ELISA kit (R&D MFGE80). Absorbance (at 450 nm) with wavelength correction at 540 nm was measured on the Tecan plate reader.

Interaction analysis

The MosaicIA - Interaction Analysis toolkit in ImageJ (http://mosaic.mpi-cbg.de/?q=downloads/imageJ) was used to analyze the strength of interaction between neurons and microglia in the facial nucleus. For proximity analysis, sections were imaged under a 10x objective. Sections were 25 μm thick and spaced 300 μm apart. The total number of sections from 3 mice each were: C57, 23; P301S-tau 23; P301S x MFGE8 KO 19; MFGE8 KO 12.

QUANTIFICATION AND STATISTICAL ANALYSIS

Statistical analysis and graphing was performed using graph pad Prism version 7. The Kolmogorov-Smirnov test with a Bonferroni post hoc correction for multiple comparisons was applied where appropriate to analyze differences between the cumulative frequencies of AnnV intensities. Five fields of neurons per coverslip were counted from the center of the coverslip to its periphery in a blinded fashion by assigning each source of neurons a random file number. The distribution of HT7/β-III−tubulin values for 5 m P301S-tau, 2 m P301S-tau and 5 m ALZ17 mice passed the normality test (D'Agostino & Pearson, n = 21, $K^2 = 3.305$, $p = 0.1915$). The following numbers of neurons were counted: 5 m P301S DRG Naive, 2578; 5 m P301S DRG+BV2, 2431; 5 m P301S DRG+ BV2/transwell 2270; 2 m P301S DRG Naive, 2202; 2 m P301S DRG+BV2 1523; 2 m P301S DRG+BV2/transwell 3524; 5 m ALz17 DRG Naive 1956; 5 m ALz17 DRG+BV2 1604; 5 m ALz17 DRG+BV2/transwell 2377; C57 DRG Naive 2099; 5 m C57 DRG+BV2 2236; 5 m C57 DRG+BV2/transwell 2010. All other samples were compared by Students t test, 1-way or 2-way analysis of variance (ANOVA) followed by an appropriate post hoc test as indicated. Significant differences are reported as *$p < 0.05$, **$p < 0.01$, ***$p < 0.001$, ****$p < 0.0001$.

Cell Reports, Volume *24*

Supplemental Information

Living Neurons with Tau Filaments

Aberrantly Expose Phosphatidylserine

and Are Phagocytosed by Microglia

Jack Brelstaff, Aviva M. Tolkovsky, Bernardino Ghetti, Michel Goedert, and Maria Grazia Spillantini

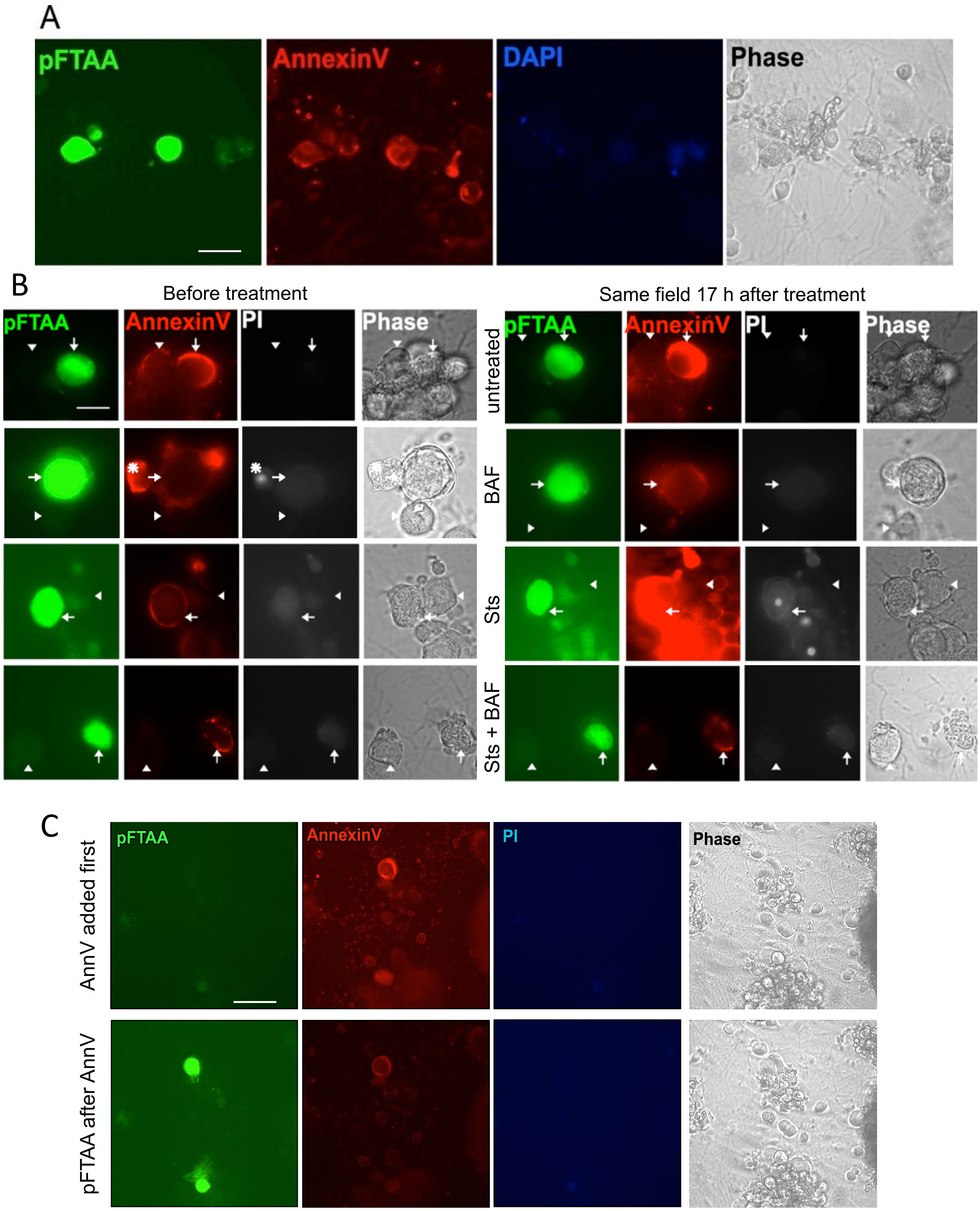

Figure S1. Neurons with tau aggregates are living neurons. Related to Figure 1.
(A) Living pFTAA+ve neurons displaying PtdSer are not stained with the live-cell-impermeable nuclear stain DAPI.
(B) The pan-caspase inhibitor Boc-Asp-FMK (BAF) prevents neurons treated with 250 nM Staurosporine (Sts) for 17 hours from displaying PtdSer during apoptosis and becoming PI+ve due to secondary necrosis, but it has no effect on PtdSer exposure in pFTAA+ve neurons.
(C) AnnV staining of living neurons with tau aggregates is enhanced independently of pFTAA binding. Scale bars: A = 50 µm, B = 25 µm, C=50 µm.

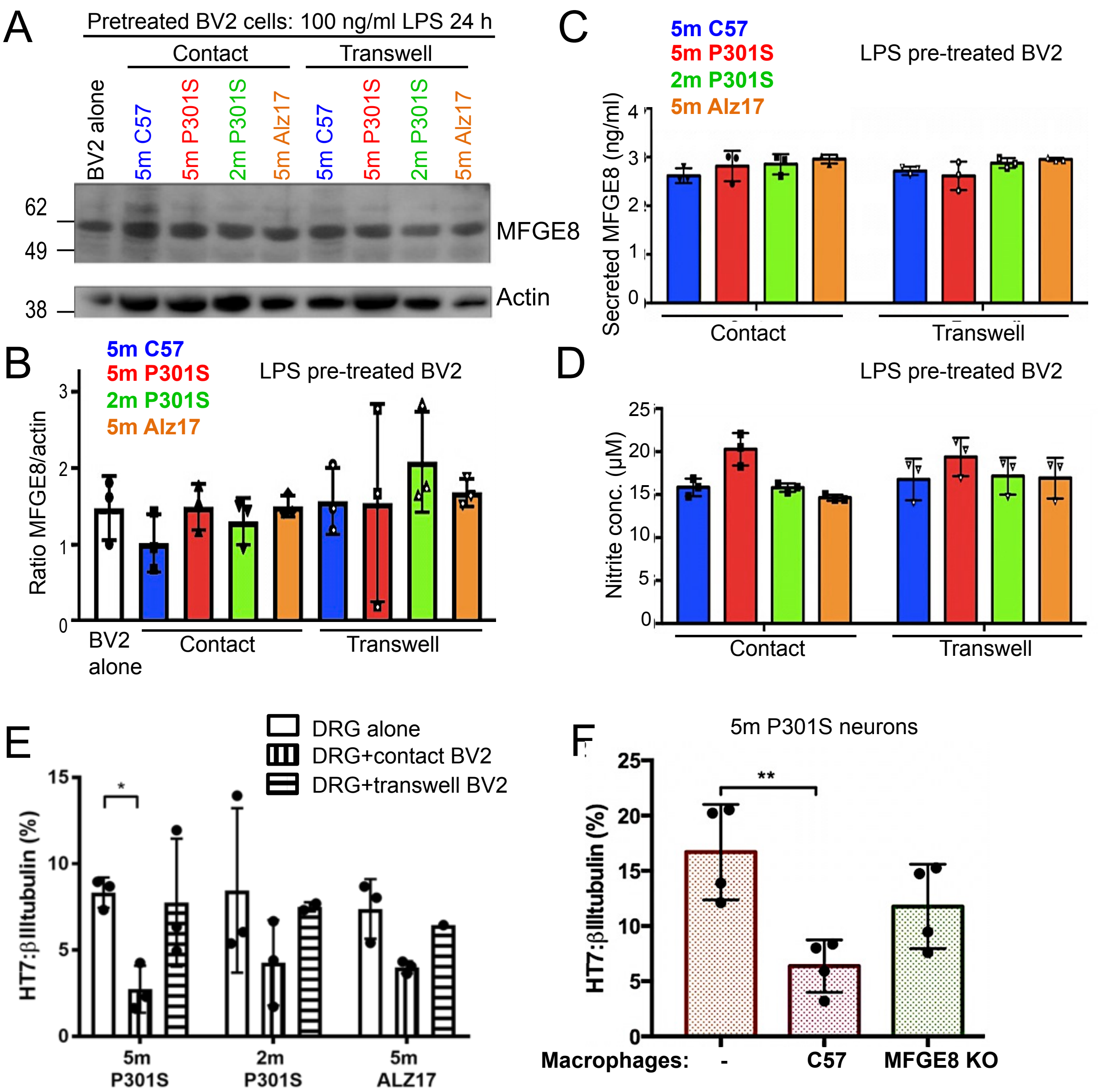

Figure S2. LPS pretreated BV2 cells produce the same amounts of MFGE8 and NO irrespective of neuron source or culture conditions but still favour 5m P301S-tau+ve neurons for phagocytosis. Related to Figure 3.

(A) Representative immunoblot showing that BV2 cells treated with 100 ng/ml LPS for 24 h and washed before culturing alone or in co-culture with neurons for 4 days express similar elevated amounts of MFGE8 irrespective of neuronal source and regardless of whether the BV2 cells were in contact with the neurons or in transwells.

(B) Quantification of MFGE8 expression from 3 independent experiments.

(C) The amount of MFGE8 secreted from BV2 cells pretreated with 100 ng/ml LPS for 24 h and co-cultured with neurons results in no differences, as described in A.

(D) Treatment of BV2 microglia with LPS as described in (A) induced similar amounts of NO (around 15 μM nitrite equivalents) to those induced by neurons from 5m P301S-tau mice.

(E) The percentage of HT7+ve neurons vs. total βIIItubulin+ve neurons remaining after neurons were cultured with LPS-pre-treated BV2 microglia in contact or via transwells for 4 days. DRG neurons from 5m P301S-tau mice are lost from cultures in contact with LPS pre-treated BV2 microglia (*=$p<0.05$) but not when BV2 microglia are in transwells. No HT7+ve neurons are lost when BV2 microglia are cultured with neurons from 2m P301S-tau mice or 5m Alz17 mice. Mean ± SD, n=3 independent experiments. 2-way ANOVA, Bonferroni corrected.

(F) Bone marrow-derived macrophages (BMDM) cultured from C57 mice in contact with DRG neurons from 5m P301S-tau mice induced significant loss of htau+ve neurons ($p<0.01$), but there was no significant loss of neurons when BMDMs were prepared from MFGE8 knockout mice, 1-way ANOVA, Bonferroni corrected.

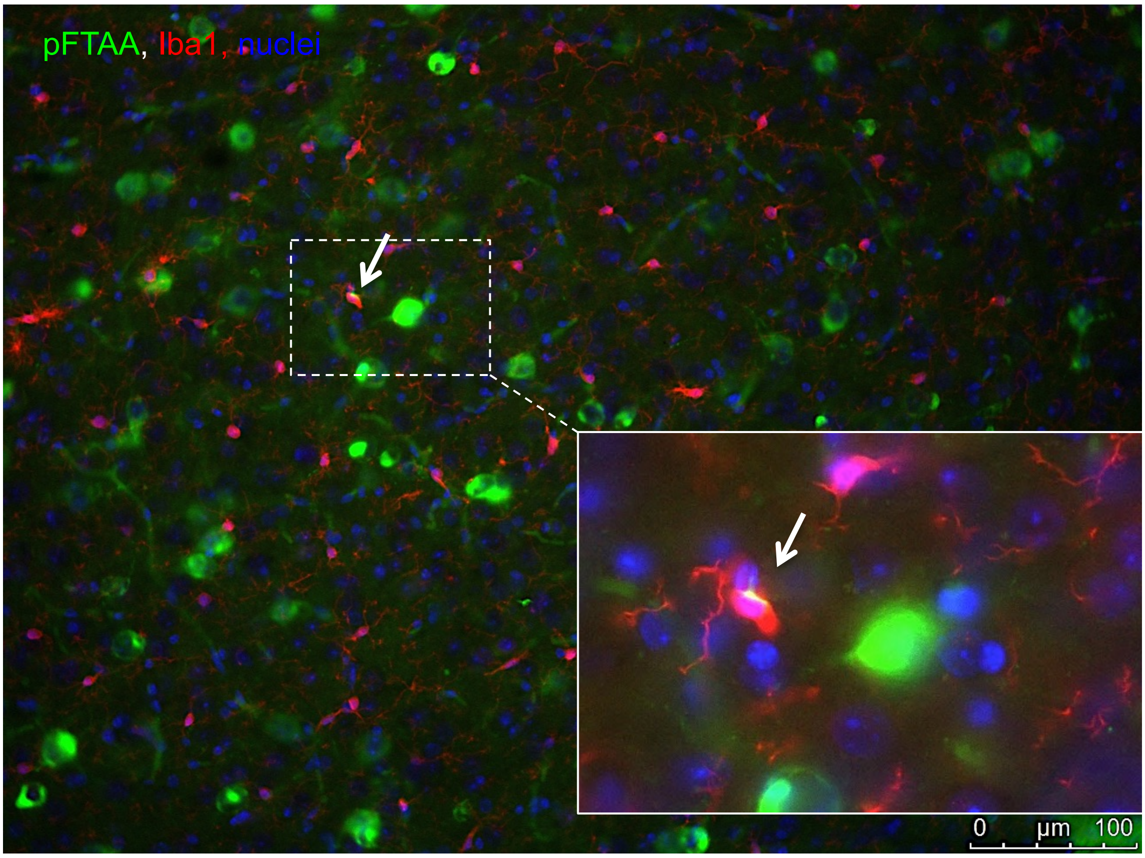

Figure S3. pFTAA+ve tau inside a microglial cell in the motor cortex. Related to Figure 4.
Section (25 µm thick) through the level of the primary motor cortex M1 (interaural 5.12 mm, Bregma 1.32 mm) stained with pFTAA (green), anti-Iba1 (red), and Dapi (blue).

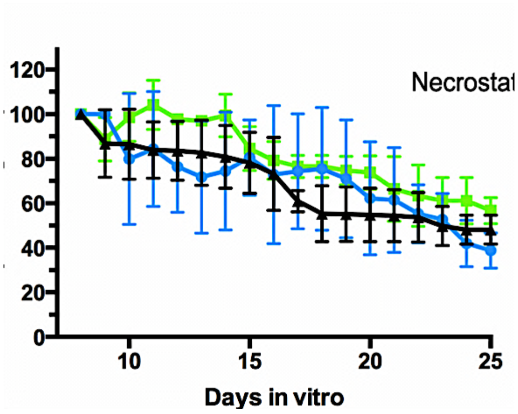

Figure S4. Loss of P301S-tau neurons is not due to necroptosis. Related to Figure 4.

The rate of pFTAA+ve neuron loss is not altered when DRG cultures from 5m P301S-tau are maintained in the presence of 10 μM necrostatin-1 (Nec1) or 10 μM of the inactive analogue (Nec1i). DRG neurons from 5m P301S-tau mice were maintained in culture for 8 days before addition of the inhibitors. Each day, the number of pFTAA +ve neurons remaining in the same fields was counted. Each point shows mean ± SD, n=3 independent experiments.

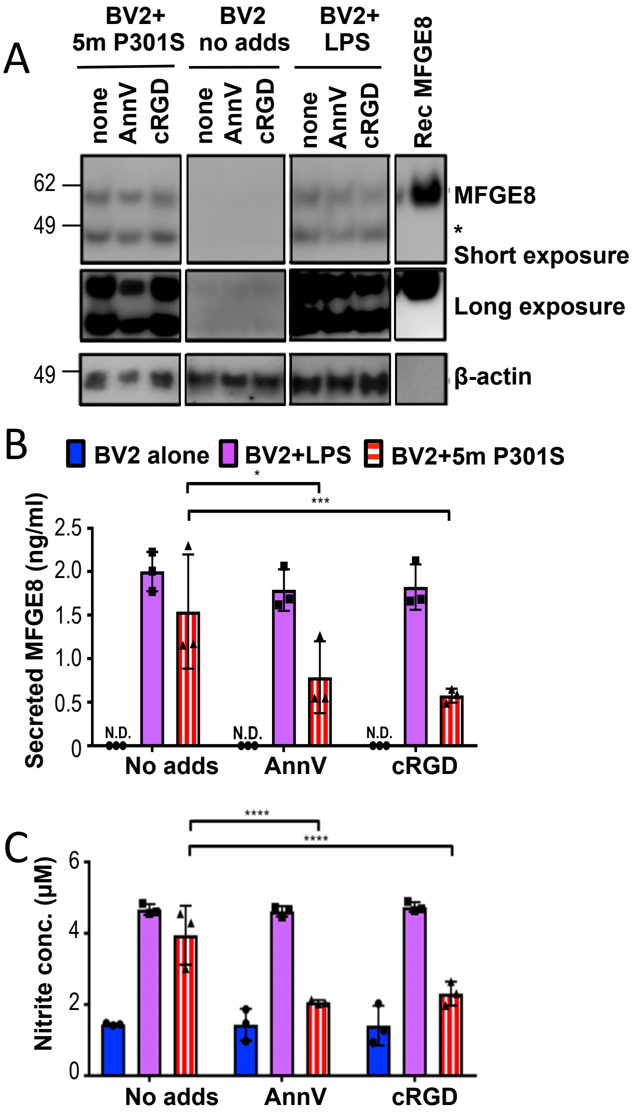

Figure S5. AnnV or RGD do not inhibit MFGE8 or NO release from LPS-treated BV2 cells. Related to Figure 5.

(A-C) AnnV or cRGD do not inhibit MFGE8 expression (D), MFGE8 secretion (E) or NO production (F) by BV2 cells stimulated with LPS, nor inhibit the intracellular amounts of MFGE8 expressed when BV2 cells are cultured with neurons from 5m P301S-tau mice. Both drugs do inhibit MFGE8 and NO secretion in the co-cultures. * indicates either another isoform of MFGE8 or a breakdown product. Mean ± SD, n=3 independent experiments analysed by 1-way ANOVA, Bonferroni corrected.
